# Evaluation and refinement of thresholds for early migration of total knee replacements as an estimator of late aseptic loosening: an updated systematic review of RSA and survival studies

**DOI:** 10.2340/17453674.2024.42574

**Published:** 2025-01-07

**Authors:** Raymond PUIJK, Jiwanjot SINGH, Rowan H PUIJK, Elise K LAENDE, José W M PLEVIER, Peter A NOLTE, Bart G C W PIJLS

**Affiliations:** 1 Department of Orthopaedics, Spaarne Gasthuis, Hoofddorp, the Netherlands; 2 Mechanical and Materials Engineering, Queen’s University, Kingston, Ontario, Canada; 3 Division of Orthopaedics, Department of Surgery, Dalhousie University and QEII Health Sciences Centre, Nova Scotia Health Authority, Halifax, Nova Scotia, Canada; 4 Walaeus Library, Leiden University Medical Center, Leiden, The Netherlands; 5 Department of Oral Cell Biology, Academic Centre for Dentistry (ACTA), University of Amsterdam and Vrije Universiteit Amsterdam, Amsterdam, the Netherlands; 6 Department of Orthopaedics, Leiden University Medical Center, Leiden, The Netherlands

## Abstract

**Background and purpose:**

This study updates 2 parallel systematic reviews and meta-analyses from 2012, which established the 1-year radiostereometric (RSA) migration thresholds for tibial components of total knee replacements (TKR) based on the risk of late revision for aseptic loosening from survival studies. The primary aim of this study was to determine the (mis)categorization rate of the 2012 thresholds using the updated review as a validation dataset. Secondary aims were evaluation of 6-month migration, mean continuous (1- to 2-year) migration, and fixation-specific thresholds for tibial component migration.

**Methods:**

One review comprised early migration data, measured by maximum total point motion (MTPM), from RSA studies, while the other focused on revision rates for aseptic loosening of tibial components from survival studies. Studies were matched based on prosthesis, fixation (i.e., cemented and uncemented, and uncemented with screw fixation), and insert (PFI). For the primary aim, newly included study group combinations were compared with the 2012 RSA thresholds to determine the (mis)categorization rate. For the secondary aims, new thresholds were determined based on revision rates for any reason in national registries (5-year < 3%, 10-year < 5%, 15-year < 6.5%).

**Results:**

After matching studies on PFI, a total of 157 survival and 82 RSA studies were included, comprising 504 study group combinations, 51 different PFIs, and 186,974 TKRs. We found that the 2012 thresholds were valid, with a misclassification rate of 0.5% at 5 and 0.3% at 10 years. Mean continuous migration could not be used to identify safe or unsafe implants. For cemented TKR, the 6-month mean MTPM was acceptable below 0.30 mm and unacceptable above 1.10 mm. For uncemented TKR, it was acceptable below 1.10 mm and unacceptable above 1.55 mm.

**Conclusion:**

The updated data reaffirm the 2012 RSA thresholds, confirming their validity in estimating revision risks for tibial component aseptic loosening. The newly proposed fixation-specific 6-month migration thresholds were found to be reliable for early identification of unsafe TKR designs, while 1- to 2-year mean continuous migration data were found not to be reliable for this purpose. These findings support and refine the migration thresholds to improve the evidence-based introduction of new TKR systems.

Since 2017, the European Union has implemented new medical device regulations mandating implant manufacturers to provide clinical evidence of whether the performance of newly introduced implants outweighs the risks to patient safety [1]. Radiostereometric analysis (RSA) has emerged as a valuable surrogate for assessing long-term outcomes of implants in a small group of patients with a follow-up of 1 year [2-5]. The effect of RSA testing can be observed in national joint registries, showing an approximate 1% decrease in the mean all-cause revision rate for total knee replacements (TKR) after 5- and 10-year follow-up among RSA-tested compared with non-RSA-tested TKRs [3]. This reduction translates to a 10–35% reduction in the total number of TKR revisions [4]. RSA has been proposed as a necessary tool in preclinical testing to provide early warnings and protect patients from newly introduced implants, fixation methods, or inserts that potentially have inferior outcomes [2,3,5,6].

Over a decade ago, the RSA and survival study meta-analysis conducted by Pijls et al. (2012) established 1-year migration thresholds for tibial components of TKR. These thresholds categorized migration as acceptable, at-risk, or unacceptable based on their corresponding revision rates at 5 and 10 years [5]. However, with the ongoing development and introduction of new TKRs, one may wonder whether the 2012 thresholds are still valid and whether they apply to modern designs including new uncemented fixations [7]. Therefore, the primary aim of this study was to determine the categorization rate of the 2012 thresholds using the updated review as a validation dataset. Secondary aims were the evaluation of thresholds based on 6-month migration, based on mean continuous migration (difference between first and second year), and fixation-specific (i.e., cemented and uncemented without screw fixation, uncemented with screw fixation) thresholds.

## Methods

We performed a 2-sided parallel systematic review of the literature for both RSA and survivorship studies on TKR tibial components, which serves as an update to the previous meta-analysis published in 2012 [5]. The reviews considered the literature for: (i) early migration of tibial components of TKR from RSA studies, and (ii) revision rates for aseptic loosening of tibial components of TKR from survival studies. The results of the reviews were matched for tibial component design, including all technical factors mentioned by studies on the prosthesis, fixation (e.g., cemented, uncemented with or without screw fixation), and insert (e.g., cruciate retaining or posterior stabilized) (PFI) [5]. The reporting of this systematic review adheres to the standards of the updated Preferred Reporting Items for Systematic Review and Meta-Analysis (PRISMA) Statement of 2020 [8] (see Supplementary data). The protocol, and its amendments, has been registered a priori in the Open Science Framework (OSF): URL https://osf.io/96bnq/?view_only=0912275f5c364fffb3eec63921cf2925 .

### Review of survival studies

*Eligibility criteria.* Studies were considered eligible based on the following criteria: (i) primary TKR; (ii) revisions or indications for revision surgery related to aseptic loosening of the tibial component as an endpoint; (iii) a follow-up duration of 5, 10, 15, 20, or 25 years; and (iv) reporting of revision percentages at the 5-year interval follow-up points. Studies were excluded if: (i) there were fewer than 75 TKRs in each treatment arm at baseline; (ii) the follow-up of a complete cohort was less than 5 years; (iii) studies were not written in English, Dutch, or German; (iv) studies lacked sufficient information on the PFI used; (v) studies lacked adequate information concerning revisions; and (vi) used a PFI that did not match any of the PFIs included in the updated RSA review. Studies that were initially excluded from the previous review due to the inability to find matching PFIs with any of the RSA studies have been reevaluated for potential inclusion.

*Search strategy.* The previous search strategy was updated by the same medical librarian (JP) for the same medical bibliographic databases (i.e., PubMed, Embase, Web-of-Science, and the Cochrane Library), with the addition of Google Scholar. To identify new results, the search was limited to publications from 2008 to 2023, with a controlled vocabulary and free-text terms related to (i) joint replacement; (ii) implant failure; and (iii) survival analysis. No differentiation between knee and hip arthroplasty was utilized, as some studies report on both [5]. The full search strategies for all databases including utilized filters are detailed in Table SM2 (see Supplementary data).

*Study selection.* After merging the records of individual databases and removal of duplicates, references were transferred to Excel (version 16.69.1, Microsoft Corp, Redmond, WA, USA), in which screening was first performed in duplicate by 2 reviewers (RP, JS) based on the title and abstract, and thereafter on the full text. A third reviewer (BP) was consulted to resolve study eligibility disagreements, and articles remained eligible for full-text screening in case of insufficient information stated in the abstract.

*Data collection, items, and synthesis.* Data extraction from 2008–2023 publications were performed in Excel (version 16.69.1, Microsoft) in duplicate by 2 reviewers (RP, JS) independently. Data extraction of articles included in the meta-analysis of 2012 was checked by 1 reviewer (RP). Any missing or unclear information was obtained or clarified by contacting the study investigators, and each item of correspondence was archived. Extracted data items included the study, patient, and implant characteristics, and revision rates for aseptic loosening of the tibial component at 5-year intervals, all similar to those of the previous meta-analysis from 2012 [5]. For each included study, study groups were categorized according to the PFI methodology [5].

### Review of RSA studies

The methodology for the literature review of RSA studies that investigated the migration of tibial components was defined in our previous meta-analysis from 2023 [7], which applied to all RSA studies published before 2023. Briefly, implant migration was recorded, quantified as mean maximum total point motion (MTPM) as this was the most commonly reported metric. The full search strategies for all databases including utilized filters are detailed in Table SM3 (see Supplementary data). Similarly, study groups were categorized according to the PFI methodology [5]. RSA studies were secondarily excluded if they investigated a PFI not matching any included in the survival study review.

### Data synthesis of combined reviews

A study group is defined as a group of patients in a study with the same PFI, and a single study can have multiple study groups. To mitigate confounding related to prosthesis factors, study groups from RSA and survival studies were matched by their shared PFI. A single PFI might have multiple “RSA-survival combinations“ based on the number of study groups with a similar PFI [5]. For instance, if 2 RSA study groups and 4 survival groups shared a PFI, this resulted in 8 combinations (i.e., 2 times 4 = 8 combinations).

### Quality assessment

To appraise the internal validity of a study, the AQUILA methodological score was used, which was specifically constructed for cohort studies regarding lower limb arthroplasty [9]. The score could vary between 11 (excellent) and 0 (poor) for survival studies, and 8 (excellent) and 0 (poor) for RSA studies [5,9]. The AQUILA score was independently assigned for each publication by 2 reviewers (JS, RHP).

The external validity across study groups was addressed by using a match score to assess the similarity of populations between RSA and survival studies [5]. The match score was based on several factors, including age, sex, diagnosis, hospital type, and continent, and could vary between 5 (excellent) and 0 (poor) [5]. The match score calculation by each item is detailed in Table SM4 (see Supplementary data). By evaluating these characteristics, we aimed to quantify and describe the degree of similarity between the populations involved in the RSA and survival study combinations, enhancing the generalizability of the findings.

### Computation of migration thresholds

The migration thresholds in this study were based on migration categories, defined as acceptable, at risk, and unacceptable early migration measured by MTPM, aligning with nationally accepted revision rate standards [5]. Because no nationally established standards exist specifically for the revision rates of aseptic loosening in tibial components, we have used nationally established revision rates for any cause as a benchmark. The rationale for this approach is that newly introduced tibial components should, at a minimum, perform as well as the current all-cause revision standards. This aligns with the goal of RSA benchmarks, which is to identify high-risk “disaster” implants that pose a significant risk of patient harm. The revision standards that are used as benchmarks are sourced from national registries for 5 years (< 3%) and 10 years (< 5%) post-surgery (NJR 2023, SKAR 2023, AJR 2023, NZJR 2023 [10-13]), and for 15 years (< 6.5%) post-surgery the standard of the Orthopaedic Data Evaluation Penal (ODEP) Rating System [14]. Similar to the previous review, we solely extracted revision rates due to aseptic loosening of the tibial component from studies for our analysis [5]. By using the revision rate standards, 3 migration categories (acceptable, at risk, and unacceptable) were determined. A migration was defined as “acceptable” when all revision rates of tibial components did not exceed the revision standard of the specific follow-up (3%, 5%, or 6.5%). Component migration was defined as “unacceptable” when all revision rates exceeded the revision standard of the specific follow-up. The “at risk” category encompasses the migration range falling between the “acceptable” and “unacceptable” thresholds, including studies with both lower and higher revision rates than the standards at a specific follow-up.

### Statistics

As the purpose of this implant safety study is to prevent unsafe implants from entering the market, the study leverages the established association between early migration, as measured by RSA, and late revision due to aseptic loosening [5,15]. Both factors are significantly influenced by the type of PFI. However, due to the challenges inherent in conducting studies that investigate both early migration and late revision, this study indirectly compares these factors using data from separate studies, similar to the descriptive method of the previous review (2012) [5]. For comparisons, scatterplots were created with revision rates (y-axis) and migration data (x-axis), alongside the calculated limits of the migration categories [5]. First, the 2012 fixation-independent 1-year migration thresholds (5 years: 0.54 and 1.60 mm; 10 years: 0.45 and 1.60 mm) were validated, by evaluating how many current study combinations were miscategorized. Additionally, the internal validity and similarity of the miscategorized study combinations were evaluated, by use of the AQUILA methodological-quality [9] and match-score, respectively. Second, the usability and meaningfulness of the mean difference between 1- and 2-year migration (Δ1–2-year) for migration thresholds was evaluated, considering all study combinations together, and only the “at-risk” categorized combinations, as proposed by Pijls et al. (2018) [16]. The Δ1–2-year MTPM was preferably obtained directly from the studies when it was reported. However, if it was not reported, we calculated this difference by subtracting the mean 1-year MTPM from the mean 2-year MTPM, both referenced to the baseline. Lastly, fixation-specific scatterplots were created and evaluated for migration thresholds, by using 6-month and 1-year migration, and 5-, 10-, and 15-year revision data. All analyses were performed with RStudio version 2023.12.0+369 (Rstudio, PBC, Boston, MA, USA) and the “ggplot2” package.

Because the 10- and 15-year data for TKRs with high revision rates may not always be published once the 5-year or 10-year results are available, we estimated the missing data to account for a potential effect of publication bias [5]. To estimate the missing data, we analyzed the average increase in revision rates for aseptic loosening from 5 to 10 years, based on the available data of studies that did report at these follow-up points. This analysis showed a multiplying factor of 1.5, indicating a 50% increase in revision rates for aseptic loosening at 10 years compared with at 5 years. As for the 15 years missing results, the same approach was performed, resulting in a multiplying factor of 1.7, indicating a 70% increase in revision rates at 15 years compared with at 10 years. Accordingly, missing values were calculated by use of these multiplying factors. To verify whether the estimated revision rates followed the correct pattern, we compared the estimated 10-year results with the actual 10-year results for the complete cases. Similarly, we compared the estimated and actual results for the 15-year data [5]. The analyses showed a minimal difference between the estimated and actual results: 0.3% (SD 1.0) for the 10-year revision rates and 0.2% (SD 1.4) for the 15-year revision rates. This indicates minimal systematic error and minimal influence on the thresholds. Also, the calculated multiplying factors were found to be comparable with the 10-year pattern of revision rates for aseptic loosening reported by the New Zealand Joint Registry (NZJR) [12]. Overall, the proportions of study combinations with estimated data were 0.0% at 5 years, 46.0% at 10 years, and 86.1% at 15 years. A breakdown of the actual and estimated data for the 3 main fixation types at the different follow-up points is provided in Table 1. As sensitivity analyses, we reported the migration thresholds when the estimated data was calculated with a multiplying factor of 1.0, representing no change in revisions after the previous follow-up.

**Table 1:** Breakdown of number of reported and estimated study combinations and implants by each follow-up mark, used for analyses (1-year migration data). Values are count (%)

Follow-up	Reported data by studies			Estimated data			Final data used ^a^
	5 years	10 years	15-years	5 years	10 years	15 years	
Cemented							
Study combinations	399 (100)	224 (54)	56 (14)	0 (0.0)	187 (45)	355 (86)	399 (100)
Implants	132,360	107,351	23,917	0	50,369	133,803	132,360
Uncemented without screw fixation							
Study combinations	82 (100)	41 (50)	12 (15)	0 (0.0)	41 (50.0)	70 (85)	82 (100)
Implants	15,163	7,324	3,548	0	7,839	11,615	15,163
Uncemented with screw fixation							
Study combinations	11 (100)	7 (64)	2 (18)	0 (0.0)	4 (36)	9 (82)	11 (100)
Implants	1,242	810	248	0	432	994	1,242

a Includes the data used for analyses, which is an accumulation of the reported and estimated data.

### Data sharing, funding and disclosure

The data extraction of the RSA and survival studies is available by contacting the corresponding author. Funding for the study was obtained from the author’s institution. BP and JP were authors of the previous systematic review and meta-analysis [5]. RP, EL, PN, and BP were part of an investigator team for 1 or more of the included studies. No author had any conflict of interest to declare. Complete disclosure of interest forms according to ICMJE are available on the article page, doi: 10.2340/17453674.2024.42574

## Results

### Included studies and matched PFIs

**Figure 1:**
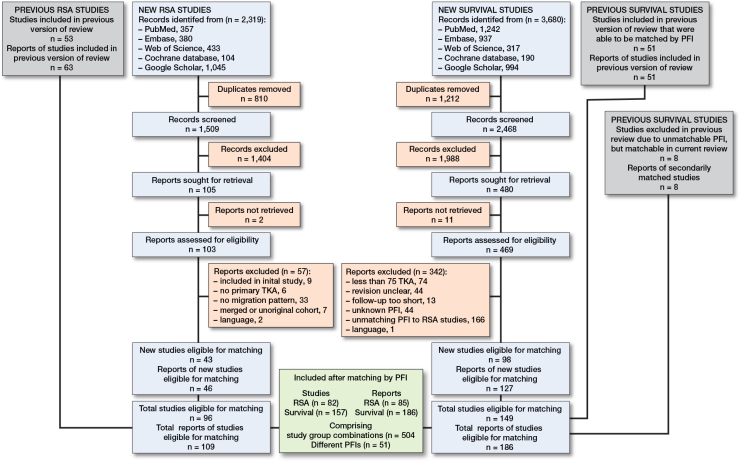
Flow diagram of articles screened, selected, included, and combined from both systematic reviews and meta-analyses. The definition of a report is that multiple (follow-up) reports can be published concerning a single study (cohort).

The inclusion of reports and studies of both meta-analyses is depicted in Figure 1. A study was defined as a study cohort, on which multiple reports (follow-up papers) could be published. For the survival review, the updated literature search yielded 3,680 records, of which 1,212 were duplicates. A total of 127 new reports, related to 98 original studies, were eligible for matching the RSA studies. Together with the previous survival study review, and studies that were previously excluded because no RSA study with a matching PFI existed, a total of 186 reports related to 149 studies were included. For the RSA review, the updated review of RSA studies included 109 reports related to 96 original studies [7]. After matching the eligible survival and RSA studies, a total of 85 RSA reports (82 studies) and 186 survival reports (157 studies) were included, comprising 504 study group combinations, and 186,974 knee arthroplasties of 51 different PFIs (Figure 1). All different PFIs that were found and matched are reported in Table 2.

**Table 2:** Prosthesis, fixation, and insert (PFI) characteristics

Prosthesis		Fixation	Insert	Number of study groups		
				RSA	survival	combined
1	ACS, PS, MB	Cemented	FB, Mod	1	1	1
2	ACS, CS, MB	Cemented	MoB, Mod	2	1	2
3	ACS, CS, MB	Porous + TiN **^a^**	MoB, Mod	1	2	2
4	Advance, CS, MB	Cemented	FB, Mod	1	6	6
5	AGC, CR, MB **^b^**	Cemented	FB non-Mod	2	5	10
6	AGC 2000, CR, MB **^b^**	Porous **^a^**	FB, non-Mod	1	2	2
7	Anatomic Modular Knee, CR, MB	Cemented	FB, Mod	3	2	6
8	Duracon, CR, MB	Cemented	FB, Mod	2	2	4
9	Duracon, CR, MB	Porous + PA **^a^**	FB, Mod	4	1	4
10	Freeman-Samuelson, CR	Uncoated **^a^**	All-poly (HDP)	2	2	4
11	Freeman-Samuelson, PE pegs, CR, MB	Uncoated **^a^**	FB, non-Mod	2	1	2
12	Freeman-Samuelson, metal pegs, CR, MB	Cemented	FB, Mod	2	2	4
13	Freeman-Samuelson, PE pegs, CR, MB	Cemented	FB, non-Mod	1	2	2
14	Genesis II, PS, MB	Cemented	FB, Mod	3	5	15
15	Interax, CR, MB	Uncoated **^a^**	FB, MenB	2	1	2
16	Kinematic condylar, CR, MB	Cemented	FB, non-Mod	1	7	7
17	Kinemax plus, CR,	Cemented	All-poly	1	2	2
18	LCS, CS, MB	Porous **^a^**	MB, Mod	1	11	11
19	Maxim, I-beam stem, CR, MB	Cemented	FB, Mod	1	1	1
20	Miller Galante I, CR, MB	Porous + screws **^a^**	FB, Mod	2	2	4
21	Miller Galante I, CR, MB	Cemented	FB, Mod	1	2	2
22	Miller Galante II, CR, MB	Cemented	FB, Mod	2	1	2
23	Miller Galante II, CR, MB	Porous + screw **^a^**	FB, Mod	3	1	3
24	NexGen CR, MB	Cemented	FB, Mod	5	12	60
25	NexGen CR flex, MB	Cemented	FB, Mod	2	5	10
26	NexGen legacy PS, MB	Cemented	FB, Mod	4	10	40
27	NexGen legacy PS flex, MB	Cemented	FB, Mod	2	15	30
28	NexGen legacy PS flex, MB	Cemented	MoB, Mod	2	4	8
29	NexGen option stemmed, CR, MB	Cemented	FB, Mod	3	2	6
30	NexGen monoblock Legacy, PS, MB	TM a	FB, non-Mod	4	4	16
31	NexGen monoblock, CR, MB	TM a	FB, non-Mod	5	3	15
32	Optetrak, PS, MB	Cemented	FB, Mod	1	2	2
33	Porous Coated Anatomic, CR, MB	Porous + screw **^a^**	FB, Mod	4	1	4
34	Porous Coated Anatomic, CR, MB	Cemented	FB, Mod	1	1	1
35	Profix, CR, MB	Cemented	FB, Mod	6	1	6
36	Profix, CR	Cemented	All-poly	1	3	3
37	Persona, PS, MB	Cemented	FB, Mod	2	2	4
38	Persona, CR, MB	Cemented	FB, Mod	2	1	2
39	Press Fit Condylar, CR, MB	Cemented	FB, Mod	1	10	10
40	Press Fit Condylar, CR, MB	Porous **^a^**	FB, Mod	1	1	1
41	Press Fit Condylar Sigma, CR, MB	Cemented	FB, Mod	3	11	33
42	Press Fit Condylar Sigma, CR, MB	Cemented	MoB, Mod	1	4	4
43	Total Condylar, PS	Cemented	All-poly	1	5	5
44	Triathlon cruciform, CR, MB	Cemented	FB, Mod	10	5	50
45	Triathlon cruciform, PS, MB	Cemented	FB, Mod	5	3	15
46	Triathlon cruciform, PS, MB	Porous + PA **^a^**	FB, Mod	2	1	2
47	Triathlon tritanium, CR, MB	TM **^a^**	FB, Mod	4	4	16
48	Tricon M, PE pegs, MB	Porous **^a^**	FB, non-Mod	3	1	3
49	Vanguard complete, CR, MB	Cemented	FB, Mod	8	7	56
50	Vanguard complete, CR, MB	Porous **^a^**	FB, Mod	1	2	2
51	Vanguard XP, MB	Cemented	FB, Mod	2	1	2
Total				127	183	504

a Uncemented fixation, surface modification specified.

b AGC = Anatomic Graduated Component

CR = cruciate retaining; PS = posterior stabilized; CS = condylar stabilized; MB = metal backed; PE = polyethylene; TM = trabecular metal; TiN = titanium-nitride; HA = hydroxyapatite; PA = periapatite; FB = fixed bearing; MoB = mobile bearing; Mod = modular; MenB = meniscal bearings.

The mean AQUILA methodological-quality score was 5.3 (SD 1.3) on an 8-point scale for the RSA studies, and 6.6 (SD 1.5) on an 11-point scale for the survival studies. The mean match-score of RSA survival combinations was 1.9 (SD 1.1) on a 5-point scale. All included studies were separately referenced in alphabetical order in the supplementary file (see Supplementary data).

### Evaluation of the 2012 thresholds based on 1-year MTPM

**Figure 2:**
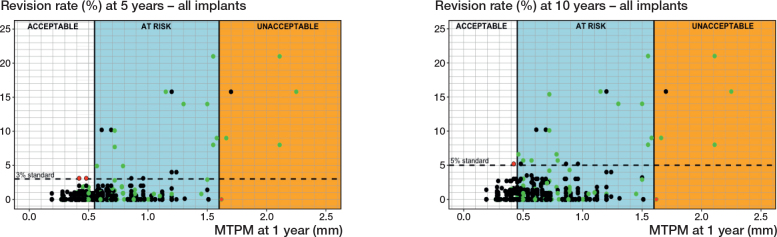
Scatterplots presenting the 2012 migration thresholds for 5 years (left panel) and 10 years (right panel) post-surgery, including the 3 migration categories (acceptable, at risk, unacceptable) and all 504 study group combinations. The 2012 migration thresholds were defined at 0.55 and 1.60 mm (5 years) and at 0.45 and 1.60 mm (10 years) [5]. Green dots indicate previously included study combinations, black dots newly included study combinations. Red dots indicate study combinations that were miscategorized by the 2012 thresholds.

Of the 415 newly included study combinations, 2 (0.5%) at 5 years and 1 (0.3%) at 10 years were misclassified based on their 1-year MTPM when using the 2012 thresholds for estimating the risk of aseptic loosening of the tibial component (Figure 2). The 2012 thresholds (based on the 1-year MTPM and 5- and 10-year revision rates of 89 study combinations) [5] are displayed for clarity (Figure 2). One study combination (Figure 2; at 5 and 10 years with a 0% revision rate and 1.63 mm migration at 1 year) was incorrectly categorized as unacceptable, instead of at-risk, in the 2012 study, despite having a 0% revision rate. Consequently, this miscategorized study combination was excluded when calculating the miscategorization rate to validate the 2012 1-year migration thresholds.

### Threshold based on the mean Δ 1–2-year continuous migration

**Figure 3:**
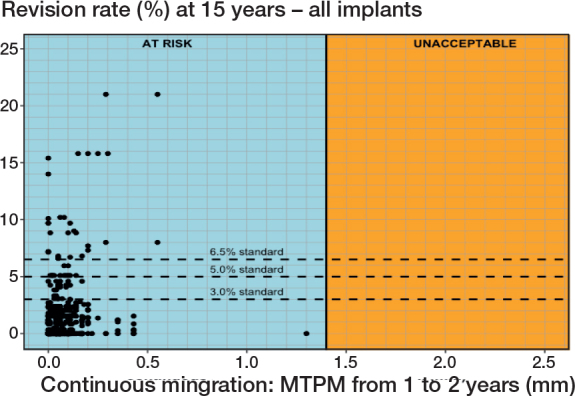
Scatterplot presenting no correlation between the mean Δ 1–2-year MTPM and revision of the tibial component for aseptic loosening at 10 and 15 years. All study combinations are considered in the figure.

When considering all tibial components together, no relationship was found between the mean magnitude of migration during the second postoperative year and the revision rate for follow-ups at 5, 10, or 15 years postoperatively. Despite exhibiting low mean continuous migration values, many tibial components still presented a heightened risk of revision due to aseptic loosening, as evidenced by the revision rates observed at the 15-year point (Figure 3). The lack of correlation held true regardless of whether all study group combinations or only the combinations with an at-risk 1-year MTPM were considered.

### Fixation-specific migration thresholds

**Figure 4:**
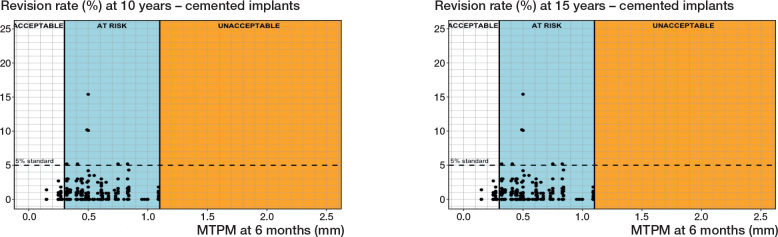
Relation between MTPM at 6 months and the revision rate of cemented tibial components for aseptic loosening at 10 years (left panel), and 15 years (right panel) including the 3 migration categories (acceptable, at risk, unacceptable) and corresponding thresholds at 0.30 and 1.10 mm.

*Cemented fixation.* 34 different PFIs were found in 411 RSA-survival study combinations that included 156,920 cemented tibial components (Tables 1 and 2). Based on a 6-month MTPM, the revision rate for aseptic loosening at 5, 10, and 15 years did not exceed 3%, 5%, and 6.5%, respectively, when components migrated less than 0.30 mm. However, a 6-month migration of more than 1.10 mm delineated the at-risk and unacceptable migration range for all 3 follow-up moments (Table 3). This resulted in cemented thresholds being established at 0.30 mm and 1.10 mm (Table 3, Figure 4). Based on the 1-year MTPM, these thresholds were slightly higher at 0.40 mm and 1.15 mm for 5-, 10-, and 15-year follow-ups.

**Table 3:** Overview of migration category thresholds in mm

	5 years		10 years		15-year	
	Lower	Upper	Lower	Upper	Lower	Upper
1-year migration thresholds						
2012 thresholds **^a^**	0.54	1.60	0.45	1.60	NA	NA
Overall	0.40	1.65	0.40	1.65	0.40	1.65
Cemented	0.40	1.15	0.40	1.15	0.40	1.15
Uncemented without screw fixation	1.15	1.65	1.30	1.65	1.10	1.65
Uncemented with screw fixation	0.56	1.10	0.56	1.10	0.56	1.10
6-months migration thresholds						
Overall	0.30	1.65	0.30	1.65	0.30	1.65
Cemented	0.30	1.10	0.30	1.10	0.30	1.10
Uncemented without screw fixation	1.10	1.55	1.15	1.55	1.10	1.55
Uncemented with screw fixation	0.56	1.00	0.56	1.00	0.56	1.00

a Based on the thresholds defined in the previous meta-analysis from 2012 [5].

Lower = threshold between acceptable and at risk;

Upper = threshold between at risk and unacceptable; NA = not available.

When estimated data was calculated with a factor of 1.0 compared with the previous follow-up, both 6-month and 1-year migration thresholds for at-risk implants would change to 1.15 mm, instead of 1.10 mm, for 15 years postoperatively only.

**Figure 5:**
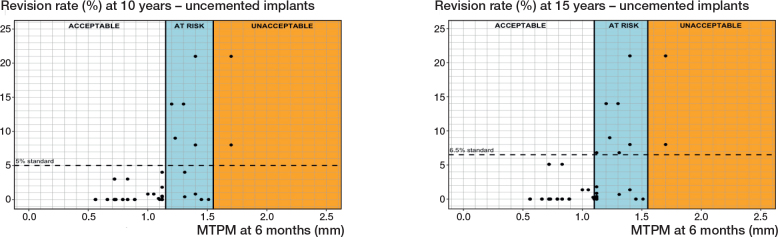
Relation between MTPM at 6 months and the revision rate of uncemented tibial components for aseptic loosening at 10 years (left panel), and 15 years (right panel) including the 3 migration categories (acceptable, at risk, unacceptable) and corresponding thresholds at 1.20 and 1.55 mm (10 years) and at 1.10 and 1.55 mm (15 years).

*Uncemented fixation without screw fixation.* 14 different PFIs were found in 82 RSA-survival study combinations that included 15,163 uncemented tibial components without screw fixation (Tables 1 and 2). Based on a 6-month MTPM, the revision rates for aseptic loosening were below 3%, 5%, or 6.5% when components migrated below 1.10 mm for all 3 follow-up points (5, 10, and 15 years). However, at all 3 follow-up moments, the revision rates exceeded the standards when the migration surpassed 1.55 mm. These findings establish 6-month migration thresholds for uncemented tibial components to be at 1.15 mm and 1.55 mm (Table 3, Figure 5). For the 1-year MTPM, these thresholds were slightly higher than those based on the 6-month MTPM (Table 3).

As for the sensitivity analysis, the 6-month migration thresholds for at-risk implants would be 1.15 mm, instead of 1.10 mm, for 15 years postoperatively only. The 1-year thresholds would not change.

**Figure 6:**
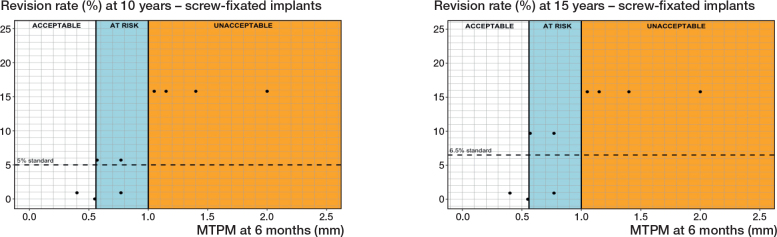
Relation between MTPM at 6 months and the revision rate for aseptic loosening of uncemented tibial components with screw fixation at 10 years (left panel), and 15 years (right panel) including the 3 migration categories (acceptable, at risk, unacceptable) and corresponding thresholds at 0.56 and 1.00 mm at 10 and 15 years.

*Uncemented with screw fixation.* 3 different PFIs were found in 11 RSA-survival study combinations that included 1,242 uncemented components with screw fixation (Tables 1 and 2). Regarding the 6-month MTPM, revision rates did not exceed 3%, 5%, or 6.5% when the component migration was below 0.56 mm. However, the revision rates were not below 3%, 5%, or 6.5% when the component migration was higher than 1.00 mm (Figure 6, Table 3). For the 1-year MTPM, these thresholds were found at 0.56 mm and 1.10 mm after 5, 10, and 15 years (Table 3).

Regarding the sensitivity analysis, no changes in the thresholds were observed for the 10-year follow-up. For the 15-year follow-up, an at-risk category could not be determined, resulting in similar thresholds of 1.00 mm after 10 years and 1.10 mm after 15 years. However, these thresholds would delineate the acceptable and unacceptable categories.

## Discussion

Our study aimed to evaluate the 2012 tibial component 1-year migration thresholds, which were defined to provide early identification of unsafe TKRs [5] against more recent migration and survivorship data. The results from our updated review ratify the 2012 migration thresholds with miscategorization rates of less than 1% on 415 newly included study combinations. We also explored alternative metrics such as 6-month migration, mean continuous migration, and fixation-specific migration thresholds. We found that it was possible to identify safe and unsafe implants based on 15-year follow-up using 6-month migration data. Fixation-specific thresholds made these predictions more precise by left-shifting the “at-risk” category for cemented implants and converging the limits of the “at-risk” category for uncemented implants. The difference in the change of limits for both fixations is most likely due to combining the fixations for threshold determination in the previous study. Specifying both fixations is important, especially for uncemented tibial components, given the renewed interest in their use.

For our first objective, the 2012 thresholds for acceptable, at risk, and unacceptable 1-year MTPM migration based on the original 89 study group combinations performed very well overall with the additional 415 study group combinations. The 2 miscategorized study combinations (i.e., i. Dunbar et al., 2009 [17] – Pulido et al., 2015 [18] 5-year thresholds; and ii. Laende et al., 2022 [19] – Pulido et al., 2015 [18] for the 5- and 10-year thresholds) of new data relative to the original 2012 thresholds had no obvious reasons underlying the misclassifications. Both combinations employed a similar PFI, the NexGen legacy posterior stabilized, metal-backed, cemented, fixed-bearing, modular TKR design. In the survival study by Pulido et al. [18] 135 patients were included, with 3.1% and 7.4% of the tibial components revised for aseptic loosening after 5 and 10 years, respectively. However, specific reasons for the revisions were not reported. The RSA study by Dunbar et al. [17] analyzed 21 TKRs over 2 years, with baseline migration analysis performed within the first 4 days postoperatively. They reported a mean 1-year MTPM of 0.48 mm. The RSA study by Laende et al. [19] involved the analysis of 9 TKRs over 2 years, with baseline analysis conducted immediately postoperatively, and a reported mean 1-year MTPM of 0.42 mm. Neither RSA study reported revisions for aseptic loosening. The AQUILA methodological-quality [9] scores were 4 and 8 for the survival studies and 4 for both the RSA studies, respectively, with a match score of 3 for both combinations. Ultimately, the miscategorized combinations slightly exceeded the threshold limits, which raises the question of whether this deviation was due to magnified migration or random variation.

Regarding our second objective, the 6-month thresholds were similar to the 1-year migration thresholds. The minimal differences observed between the 6-month and 1-year migration thresholds suggest that 6-month migration assessment may offer comparable accuracy, but has earlier detection capabilities than 1-year assessment. These findings support the recommendations of the study of Pijls et al. (2018) and Puijk et al. (2023) [7,16]. Furthermore, the fixation-specific thresholds found in this study could help give manufacturers and researchers more guidance and healthcare institutions more certainty on the performance of their TKR. Additionally, our study did not find suitability of the mean continuous migration (Δ 1–2-year) identified at-risk implant groups in RSA threshold testing. The concept of utilizing continuous migration and associated thresholds for a phased introduction of implants was initially proposed by Ryd et al. (1995) and confirmed by Molt et al. (2016) [20,21]. They found that an individual implant carries an 85% predictive risk of mechanical loosening within 10 years, when it surpasses a Δ 1–2-year MTPM of 0.1 mm/year [20]. While the Δ 1–2-year MTPM is effective at the individual level, it is not suitable for group-level risk assessment. This is likely because an increased Δ 1–2-year MTPM in 1 or 2 patients has minimal impact on the overall cohort’s mean Δ 1–2-year MTPM. As previously mentioned by Ryd et al. [20], the migration pattern of implants will likely play a substantial role in the future of implant safety, as this metric considers early migration as well as continuous migration. For future studies, the “at risk” group requires more effort to narrow down the risk of loosening, perhaps by measuring inducible displacement, as it directly measures the fixation of the implant. To consolidate all thresholds in this study, we recommend that the phased introduction of new implants should include fixation-specific thresholds at 6 months and 1 year.

### Limitations

First, due to the methodological approach of using 2 types of studies, individual patient variations are less accounted for, which can potentially lead to an aggregation bias. Individual patient and study variability could affect the accuracy of group-level conclusions [22]; unfortunately we were only able to descriptively estimate the RSA thresholds, and were not able to conduct statistical inferential methods, or consider individual patient data, due to the design of our study. Therefore, it is very important to consider that the study’s purpose is to identify unsafe implants and thus should be interpreted on a group level, rather than on an individual level [23]. Therefore, the reported thresholds should not be attributed to the implant’s performance on an individual patient level. Besides, as the migration and revision rates were sourced from different studies, migration data were not available in the survival studies to influence the decision to perform a revision. Therefore, our results do not suffer from an incorporation bias.

Second, the nationally established revision rate benchmarks used in this study considered revision rates for any cause, while the extracted data focused solely on rates for aseptic loosening of the tibial component. Ideally, benchmark rates for aseptic loosening of tibial components would have been used, to more accurately estimate their long-term revision risk for aseptic loosening based on migration. However, the approach used in this study is still effective in identifying disaster implants that pose a significant risk of patient harm.

Third, we estimated future revision rates based on reported data from previous follow-up periods. While this approach may lead to less accurate thresholds, it accounts for publication bias, which could affect the results more than the data estimation. Therefore, we performed sensitivity analyses that demonstrated nearly equal thresholds, indicating that the data estimation followed a correct pattern. Additionally, it also accounted for a potential selection bias, as the available reported data would represent less data on modern implants compared with older implants, if no estimations were conducted.

Fourth, the study included separate migration thresholds for uncemented components with screw fixation, although with a much lower number of data points compared with the other fixation-specific thresholds. Although this may result in less accurate and reliable thresholds, they were found to lie between the cemented and uncemented thresholds, which is consistent with the results of our previous meta-analysis indicating that the early migration of screw-fixated implants falls between fully cemented and uncemented implants [7].

Fifth, our study may face attrition bias due to potential revisions of high-migrating implants between 1 and 2 years postoperatively, possibly resulting in missing data at the 2-year mark and underestimating mean continuous migration. Additionally, if studies did not specifically report the 1- to-2-year migration, this was calculated, which could mean the 2 follow-up moments represent different locations and directions of migration, introducing less accurate results. However, it is unlikely that these factors influenced our results significantly. High-migrators could only minimally alter the average Δ 1–2-year migration data. Also, as MTPM represents the greatest motion vector, which mainly occurs in markers experiencing the greatest biological effect, it is likely the same markers were used at both follow-up moments. Lastly, our study did not account for other migration parameters such as translations in directions and rotations, which are also considered predictors of aseptic loosening. However, inconsistent reporting of these parameters led to a focus solely on the MTPM.

### Conclusion

This study reaffirms the validity of the 2012 RSA thresholds for predicting revision risk due to aseptic loosening of tibial components up to 15 years, as the majority of the newly included data was correctly categorized. Additionally, the results support the use of our newly proposed fixation-specific 6-month migration thresholds for early identification of unsafe TKR designs, while highlighting that mean continuous migration from 1 to 2 years is less reliable for such estimations.

In perspective, the implementation of the 6-month fixation-specific migration thresholds could enhance the evidence-based introduction of new TKR designs, fixations, and inserts by providing early identification of unsafe TKRs. Nevertheless, these estimations might carry some inherent uncertainties and potential sources of bias.

### Supplementary data

Search strategy and references for the included RSA and survival studies can be found as supplementary data on the article page, doi: 10.2340/17453674.2024.42574

## Supplementary Material



## Notes

RP and BGP conceived the study. JWP designed the search strategy for the literature search. RP and JS performed the study selection and data extraction for the survival review, and RP and RHP did so for the RSA review. RP performed the analyses. JS and RHP and BGP ensured the accuracy of data extraction. RP, EKL, PAN, and BGP helped interpret the results. RP wrote the manuscript.

Handling co-editors: Li Felländer-Tsai and Philippe Wagner

*Acta* thanks Johan Kärrholm and Leif Ryd for help with peer review of this manuscript.
