# Supplementary material for: Evaluation and refinement of thresholds for early migration of total knee replacements as an estimator of late aseptic loosening: an updated systematic review of RSA and survival studies
Source: Acta Orthop. 2025 Jan 7;96:1–10. doi: 10.2340/17453674.2024.42574 (PMC11706017; doi:10.2340/17453674.2024.42574)
Supplement: Supplementary file 1 [file ActaO-96-42574-s1.pdf]

Table SM2. Details of the literature search strategy for survival studies.

**PubMed**<br>("Joint Prosthesis"[Mesh:NoExp] OR "Hip Prosthesis"[Mesh] OR "Knee Prosthesis"[Mesh] OR "hip prosthesis"[tiab] OR "knee prosthesis"[tiab] OR "hip prostheses"[tiab] OR "knee prostheses"[tiab] OR "prosthetic hip"[tiab] OR "prosthetic knee"[tiab] OR "TKA"[tiab] OR "THA"[tiab] OR "THR"[tiab] OR "TKR"[tiab] OR "joint replacement"[tiab] OR "Arthroplasty, Replacement"[mesh:NoExp] OR "total knee replacement"[tiab] OR "total hip replacement"[tiab] OR "total knee arthroplasty"[tiab] OR "total hip arthroplasty"[tiab] OR "Arthroplasty, Replacement, Hip"[Mesh] OR "Arthroplasty, Replacement, Knee"[Mesh]) AND ("Prosthesis Failure"[Mesh:NoExp] OR "Prosthesis Failures" [tiab] OR "Prosthesis Failure"[tiab] OR "Prosthetic Failure"[tiab] OR "Prosthetic Failures"[tiab] OR "Prosthesis Survival" [tiab] OR "Prosthesis Survivals"[tiab] OR "Prosthesis Durability" [tiab] OR "Prosthesis Durabilities" [tiab] OR "Prosthesis Loosening" [tiab] OR "Prosthesis Loosenings" [tiab] OR "prosthetic loosening" [tiab] OR "aseptic loosening" [tiab] OR "implant loosening" [tiab] OR "implant failure" [tiab] OR "Prosthesis migration"[tiab] OR "Prosthesis migrations"[tiab]) AND ("survival analysis"[MeSH:NoExp] OR "Survival Analyses" [tiab] OR "Survival Analysis"[tiab] OR "Survival Rates" [tiab] OR "Survival Rate"[tiab])<br>Filters: from 2008 - 2022/12/28
**Web of Science**<br>TS=("Joint Prosthe*" OR "hip prosthe*" OR "knee prosthe*" OR TKA OR TKR OR THA OR THR OR "hip arthroplast*" OR "knee arthroplast*" OR "knee replacement*" OR "hip replacement*") AND TS=("Prosthesis Failure*" OR "Prosthetic Failure*" OR "Prosthesis Survival*" OR "Prosthetic Survival*" OR "Prosthesis Durab*" OR "Prosthesis Loosening*" OR "prosthetic loosening*" OR "aseptic loosening*" OR "implant loosening*" OR "implant failure*" OR "Prosthesis migration*" OR "Prosthetic migration*") AND TI=(surviv*) AND PY=(2008-2023)
**Cochrane database**<br>("hip prosthesis" OR "knee prosthesis" OR "hip prostheses" OR "knee prostheses" OR TKA OR THA OR "hip arthroplasty" OR "knee arthroplasty" OR "knee replacement" OR "hip replacement"):ti,ab,kw AND ("Prosthesis Failure*" OR "Prosthetic Failure*" OR "Prosthesis Survival*" OR "Prosthetic Survival*" OR "Prosthesis Durab*" OR "Prosthesis Loosening*" OR "prosthetic loosening*" OR "aseptic loosening*" OR "implant loosening*" OR "implant failure*" OR "Prosthesis migration*" OR "Prosthetic migration*"):ti,ab,kw AND (surviv*):ti,ab,kw
**Embase**<br>(joint prosthesis/ OR exp hip prosthesis/ OR exp knee prosthesis/ OR "hip prosthe*".ti,ab. OR "hip arthroplast*".ti,ab. OR "hip replacement*".ti,ab. OR "knee prosthe*".ti,ab. OR "knee arthroplast*".ti,ab. OR "knee replacement*".ti,ab. OR "prosthetic hip*".ti,ab. OR "prosthetic knee*".ti,ab. OR "TKA".ti,ab. OR "TKR".ti,ab. OR "THA".ti,ab. OR "THR".ti,ab. OR "joint replacement*".ti,ab. OR replacement arthroplasty/ OR exp hip replacement/ OR exp knee replacement/) AND (exp prosthesis loosening/ OR "Prosthesis Failure*".ti,ab. OR "Prosthetic Failure*".ti,ab. OR "Prosthesis Survival*".ti,ab. OR "Prosthesis Durab*".ti,ab. OR "Prosthesis Loosening*".ti,ab. OR "prosthetic loosening*".ti,ab. OR "aseptic loosening*".ti,ab. OR "implant loosening*".ti,ab. OR "implant failure*".ti,ab. OR "Prosthesis migration*".ti,ab. OR "Prosthetic migration*".ti,ab.) AND (exp survival analysis/ OR "Survival Analyses".ti,ab. OR "Survival Analysis".ti,ab. OR "Survival Rate*".ti,ab.) NOT (conference OR conference abstract OR "conference review").pt. AND 2008:2023.(sa_year).
**Google Scholar**<br>"Total knee arthroplasty" AND “loosening” AND “survival” from 2008 to 2023, no citations, no patents

Table SM3. Details of the literature search strategy for RSA studies.

PubMed
(("Photogrammetry"[Mesh:NoExp] OR Radiostereometric Analysis [Mesh] OR "roentgen stereophotogrammetric analysis"[tiab] OR "RSA" [tiab] OR "Radiostereometric" [tiab] OR "Radiostereometrics" [tiab] OR "stereophotogrammetric" [tiab] OR "stereophotogrammetrics" [tiab] OR "stereophotogrammetry" [tiab] OR "stereo-photogrammetric" [tiab] OR "stereophotogrammetrics" [tiab] OR "stereophotogrammetry" [tiab] OR "Photofluorography" [Mesh] OR "roentgen fluoroscopic"[tiab] OR "roentgen fluoroscopics"[tiab] OR "roentgen fluoroscopy"[tiab])) AND (("Joint Prosthesis"[Mesh:NoExp] OR "Hip Prosthesis"[Mesh] OR "Knee Prosthesis"[Mesh] OR "hip prosthesis"[tiab] OR "knee prosthesis"[tiab] OR "hip prostheses"[tiab] OR "knee prostheses"[tiab] OR "prosthetic hip"[tiab] OR "prosthetic knee"[tiab] OR "TKA"[tiab] OR "THA"[tiab] OR "THR"[tiab] OR "TKR"[tiab] OR "joint replacement"[tiab] OR "Arthroplasty, Replacement"[mesh:NoExp] OR "total knee replacement"[tiab] OR "total hip replacement"[tiab] OR "total knee arthroplasty"[tiab] OR "total hip arthroplasty"[tiab] OR "Arthroplasty, Replacement, Hip"[Mesh] OR "Arthroplasty, Replacement, Knee"[Mesh]))
Filters: from 2008 - 2022/12/28

Web of Science
TS=("Photogrammetr*" OR "RSA" OR "radiostereometr*" OR "radio-stereometr*" OR "stereophotogrammetr*" OR "stereo-photogrammetr*" OR "roentgen fluoroscop*" OR "Photofluorograph*" OR "Photo-fluorograph*") AND TS=("Joint Prosthe*" OR "hip prosthe*" OR "knee prosthe*" OR TKA OR TKR OR THA OR THR OR "hip arthroplast*" OR "knee arthroplast*" OR "knee replacement*" OR "hip replacement*") AND PY=(2016-2023)

Cochrane database
("Photogrammetr*" OR "RSA" OR "radiostereometr*" OR "radio-stereometr*" OR "stereophotogrammetr*" OR "stereo-photogrammetr*" OR "roentgen fluoroscop*" OR "Photofluorograph*" OR "Photo-fluorograph*"):ti,ab,kw AND ("Joint Prosthe*" OR "hip prosthe*" OR "knee prosthe*" OR TKA OR TKR OR THA OR THR OR "hip arthroplast*" OR "knee arthroplast*" OR "knee replacement*" OR "hip replacement*"):ti,ab,kw

Embase
(Stereophotogrammetry/ OR exp radiostereometric analysis/ OR "RSA".ti,ab. OR "Radiostereometr*".ti,ab. OR "Radio-stereometr*".ti,ab. OR "stereophotogrammetr*".ti,ab. OR "stereo-photogrammetr*".ti,ab. OR exp fluorography/ OR "Photofluorograph*".ti,ab. OR "Photo-fluorograph*".ti,ab. OR "roentgen fluoroscop*".ti,ab.) AND (joint prosthesis/ OR exp hip prosthesis/ OR exp knee prosthesis/ OR "hip prosthe*".ti,ab. OR "hip arthroplast*".ti,ab. OR "hip replacement*".ti,ab. OR "knee prosthe*".ti,ab. OR "knee arthroplast*".ti,ab. OR "knee replacement*".ti,ab. OR "prosthetic hip*".ti,ab. OR "prosthetic knee*".ti,ab. OR "TKA".ti,ab. OR "TKR".ti,ab. OR "THA".ti,ab. OR "THR".ti,ab. OR "joint replacement*".ti,ab. OR replacement arthroplasty/ OR exp hip replacement/ OR exp knee replacement/) AND 2016:2023.(sa_year). NOT (conference OR conference abstract OR "conference review").pt.

Google Scholar
"Total knee arthroplasty" AND "radiostereometric" from 2016 to 2023, no citations, no patents

SM4. Match score item calculation

Age	Studies score 1 point for age if the mean age difference between matching studies is < 5 years; otherwise, 0 points if it exceeds 5 years or when it is not reported.
Gender	Studies score 1 point if the percentage difference between matching studies in females is < 10%; otherwise, 0 points if it exceeds 10% or when it is not reported.
Diagnosis	Studies score 1 point if the percentage difference between matching studies in diagnosis (osteoarthrosis) is < 10%; otherwise, 0 points if it exceeds 10% or when it is not reported.
Hospital type	Studies score 1 point if the studies were performed in similar type of hospitals (i.e. Academic, Developer, Special institute, High volume, Public); otherwise, 0 points if not or when it is not reported.
Continent	Studies score 1 point if the studies were performed on the same continent; otherwise, 0 points if not or when it is not reported.

The match score was determined by a Delphi-study among 37 independent experts (Pijls et al. 2011 (doi: 10.1186/1471-2474-12-173). The score ranges from 0 (poor) to 5 (excellent) points and is based on age, gender, diagnosis, hospital type, and continent.

References meta-analysis survival studies

1. **Ahmed I, Salmon LJ, Waller A, Watanabe H, Roe JP, Pinczewski LA et al.** Total knee arthroplasty with an oxidised zirconium femoral component: ten-year survivorship analysis. Bone Joint J 2016; 98-B: 58–64. doi: 10.1302/0301-620X.98B1.36314.
2. **Ali MS, Mangaleshkar SR.** Uncemented Rotating-Platform Total Knee Arthroplasty: A 4-Year to 12-Year Follow-Up. J Arthroplasty 2006; 21: 80–84. doi: 10.1016/J.ARTH.2005.04.018.
3. **Anderson JA, Sculco TP, Baldini A.** Patellofemoral Function After Total Knee Arthroplasty. Journal of Knee Surgery 2008; 21: 91–96. doi: 10.1055/S-0030-1247801/BIB.
4. **Ansari S, Ackroyd CE, Newman JH.** Kinematic posterior cruciate ligament-retaining total knee replacements. A 10-year survivorship study of 445 arthroplasties. Am J Knee Surg 1998; 11: 9–14. https://europepmc.org/article/med/9533047.
5. **Argenson JNA, Parratte S, Ashour A, Saintmard B, Aubaniac JM.** The outcome of rotating-platform total knee arthroplasty with cement at a minimum of ten years of follow-up. J Bone Joint Surg Am 2012; 94: 638–644. doi: 10.2106/JBJS.K.00263.
6. **Arora J, Ogden AC.** Osteolysis in a surface-cemented, primary, modular Freeman-Samuelson total knee replacement. J Bone Joint Surg Br 2005; 87: 1502–1506. doi: 10.1302/0301-620X.87B11.16586.
7. **Arthur CHC, Wood AM, Keenan ACM, Clayton RAE, Walmsley P, Brenkel I.** Ten-year results of the Press Fit Condylar Sigma total knee replacement. Bone Joint J 2013; 95-B: 177–180. doi: 10.1302/0301-620X.95B2.29695.
8. **Ashraf T, Beard DJ, Newman JH.** Symmetrical vs. asymmetrical total knee replacement—a medium term comparative analysis. Knee 2003; 10: 61–66. doi: 10.1016/S0968-0160(02)00088-1.
9. **Awwad GEH, Ahedi H, Angadi D, Kandhari V, Coolican MRJ**. A prospective randomised controlled trial of cemented and uncemented tibial baseplates: functional and radiological outcomes. Arch Orthop Trauma Surg 2023; 143: 5891–5899. doi: 10.1007/S00402-023-04831-Z.
10. **Baker PN, Khaw FM, Kirk LMG, Esler CNA, Gregg PJ.** A randomised controlled trial of cemented versus cementless press-fit condylar total knee replacement: 15-Year survival analysis. Journal of Bone and Joint Surgery - Series B 2007; 89: 1608–1614. doi: 10.1302/0301-620X.89B12.19363.
11. **Bakircioglu S, Caglar O, Tokgozoglu AM, Atilla B.** The influence of alignment in long-term functional outcome of total knee arthroplasty. Int Orthop 2022; 46: 1985–1990. doi: 10.1007/S00264-022-05420-2.
12. **Berger RA, Lyon JH, Jacobs JJ, Barden RM, Berkson EM, Sheinkop MB et al**. Problems with cementless total knee arthroplasty at 11 years followup. Clin Orthop Relat Res 2001; 392: 196–207. doi: 10.1097/00003086-200111000-00024.
13. **Berger RA, Rosenberg AG, Barden RM, Sheinkop MB, Jacobs JJ, Galante JO et al.** Long-term followup of the Miller-Galante total knee replacement. Clin Orthop Relat Res 2001; 388: 58–67. doi: 10.1097/00003086-200107000-00010.
14. **Bernard de Villeneuve F, Jacquet C, Puech S, Parratte S, Ollivier M, Argenson JN et al.** Minimum Five Years Follow-Up of Total Knee Arthroplasty Using Morphometric Implants in Patients With Osteoarthritis. J Arthroplasty 2021; 36: 2502–2509. doi: 10.1016/J.ARTH.2021.02.052.
15. **Bertin KC.** Tibial component fixation in total knee arthroplasty: a comparison of pegged and stemmed designs. J Arthroplasty 2007; 22: 670–678. doi: 10.1016/J.ARTH.2006.07.004.
16. **Bettinson KA, Pinder IM, Moran CG, Weir DJ, Lingard EA.** All-polyethylene compared with metal-backed tibial components in total knee arthroplasty at ten years. A prospective, randomized controlled trial. J Bone Joint Surg Am 2009; 91: 1587–1594. doi: 10.2106/JBJS.G.01427.
17. **Bistolfi A, Massazza G, Lee GC, Deledda D, Berchialla P, Crova M.** Comparison of fixed and mobile-bearing total knee arthroplasty at a mean follow-up of 116 months. J Bone Joint Surg Am 2013; 95(12):e83. doi: 10.2106/JBJS.L.00327.
18. **Bourne RB, Laskin RS, Guerin JS.** Ten-year results of the first 100 Genesis II total knee replacement procedures. Orthopedics 2007; 30: 83–85.
19. **Boyle KK, Nodzo SR, Ferraro JT, Augenblick DJ, Pavlesen S, Phillips MJ et al.** Uncemented vs Cemented Cruciate Retaining Total Knee Arthroplasty in Patients With Body Mass Index Greater Than 30. J Arthroplasty 2018; 33: 1082–1088. doi: 10.1016/J.ARTH.2017.11.043.
20. **Bozic KJ, Kinder J, Menegini M, Zurakowski D, Rosenberg AG, Galante JO et al.** Implant survivorship and complication rates after total knee arthroplasty with a third-generation cemented system: 5 to 8 years followup. Clin Orthop Relat Res 2005; 430: 117–124. doi: 10.1097/01.BLO.0000146539.23869.14.

21. **Breugem SJM, Linnartz J, Sierevelt I, Bruijn JD, Driessen MJM.** Evaluation of 1031 primary titanium nitride coated mobile bearing total knee arthroplasties in an orthopedic clinic. World J Orthop 2017; 8: 922. doi: 10.5312/WJO.V8.I12.922.
22. **Buechel FF.** Long-term followup after mobile-bearing total knee replacement. Clin Orthop Relat Res 2002; 404: 40–50. doi: 10.1097/00003086-200211000-00008.
23. **Buehler KO, Venn-Watson E, D'Lima DD, Colwell CW.** The Press-Fit Condylar total knee system: 8- to 10-year results with a posterior cruciate-retaining design. Journal of Arthroplasty 2000; 15: 698–701. doi: 10.1054/arth.2000.8189.
24. **Callaghan JJ, Beckert MW, Hennessy DW, Goetz DD, Kelley SS.** Durability of a cruciate-retaining TKA with modular tibial trays at 20 years. Clin Orthop Relat Res 2013; 471: 109–117. doi: 10.1007/S11999-012-2401-9.
25. **Campbell DG, Duncan WW, Ashworth M, Mintz A, Stirling J, Stevenson TM et al.** Patellar resurfacing in total knee replacement: a ten-year randomised prospective trial. J Bone Joint Surg Br 2006; 88: 734–739. doi: 10.1302/0301-620X.88B6.16822.
26. **Chana R, Shenava Y, Nicholl AP, Lusted FJ, Skinner PW, Gibb PA et al.** Five- to 8-Year Results of the Uncemented Duracon Total Knee Arthroplasty System. J Arthroplasty 2008; 23: 677–682. doi: 10.1016/J.ARTH.2007.06.002.
27. **Cho KJ, Seon JK, Jang WY, Park CG, Song EK.** Robotic versus conventional primary total knee arthroplasty: clinical and radiological long-term results with a minimum follow-up of ten years. Int Orthop 2019; 43: 1345–1354. doi: 10.1007/S00264-018-4231-1.
28. **Choi YJ, Lee KW, Kim CH, Ahn HS, Hwang JK, Kang JH et al.** Long-term Results of Hybrid Total Knee Arthroplasty: Minimum 10-years Follow-up. Knee Surg Relat Res 2012; 24: 79–84. doi: 10.5792/KSRR.2012.24.2.79.
29. **Choy WS, Yang DS, Lee KW, Lee SK, Kim KJ, Chang SH et al.** Cemented Versus Cementless Fixation of a Tibial Component in LCS Mobile-Bearing Total Knee Arthroplasty Performed by a Single Surgeon. J Arthroplasty 2014; 29: 2397–2401. doi: 10.1016/J.ARTH.2014.03.006.
30. **Cip J, Widemschek M, Luegmair M, Sheinkop MB, Benesch T, Martin A et al.** Conventional Versus Computer-Assisted Technique for Total Knee Arthroplasty: A Minimum of 5-Year Follow-up of 200 Patients in a Prospective Randomized Comparative Trial. J Arthroplasty 2014; 29: 1795–1802. doi: 10.1016/J.ARTH.2014.04.037.
31. **Cip J, Obwegeser F, Benesch T, Bach C, Ruckenstuhl P, Martin A et al.** Twelve-Year Follow-Up of Navigated Computer-Assisted Versus Conventional Total Knee Arthroplasty: A Prospective Randomized Comparative Trial. J Arthroplasty 2018; 33: 1404–1411. doi: 10.1016/J.ARTH.2017.12.012.
32. **Clark AN, Hounat A, O'Donnell S, May P, Doonan J, Rowe P et al.** Electromagnetic Navigated Versus Conventional Total Knee Arthroplasty-A Five-Year Follow-Up of a Single-Blind Randomized Control Trial. J Arthroplasty 2021; 36: 3451–3455. doi: 10.1016/J.ARTH.2021.06.007.
33. **Clayton RAE, Amin AK, Gaston MS, Brenkel IJ.** Five-year results of the Sigma total knee arthroplasty. Knee 2006; 13: 359–364. doi: 10.1016/J.KNEE.2006.06.003.
34. **Collins RA, Walmsley PJ, Amin AK, Brenkel IJ, Clayton RAE.** Does obesity influence clinical outcome at nine years following total knee replacement? J Bone Joint Surg Br 2012; 94: 1351–1355. doi: 10.1302/0301-620X.94B10.28894.
35. **Crawford DA, Berend KR, Nam D, Barrack RL, Adams JB, Lombardi AV et al.** Low Rates of Aseptic Tibial Loosening in Obese Patients With Use of High-Viscosity Cement and Standard Tibial Tray: 2-Year Minimum Follow-Up. J Arthroplasty 2017; 32: S183–S186. doi: 10.1016/J.ARTH.2017.04.018.
36. **Crockarell JR, Hicks JM, Schroeder RJ, Guyton JL, Harkess JW, Lavelle DG et al.** Total Knee Arthroplasty With Asymmetric Femoral Condyles and Tibial Tray. J Arthroplasty 2010; 25: 108–113. doi: 10.1016/J.ARTH.2008.11.002.
37. **Dalury DF, Gonzales RA, Adams MJ, Gruen TA, Trier K.** Midterm results with the PFC Sigma total knee arthroplasty system. J Arthroplasty 2008; 23: 175–181. doi: 10.1016/J.ARTH.2007.03.039.
38. **Dalury DF, Barrett WP, Mason JB, Goldstein WM, Murphy JA, Roche MW et al.** Midterm survival of a contemporary modular total knee replacement: a multicentre study of 1970 knees. J Bone Joint Surg Br 2008; 90: 1594–1596. doi: 10.1302/0301-620X.90B12.21064.
39. **DeFrancesco CJ, Canseco JA, Nelson CL, Israelite CL, Kamath AF.** Uncemented tantalum monoblock tibial fixation for total knee arthroplasty in patients less than 60 years of age mean 10-year follow-up. J Bone Joint Surg Am 2018; 100: 865–870. doi: 10.2106/JBJS.17.00724.

40. **Dehl M, Bulaïd Y, Chelli M, Belhaouane R, Gabrion A, Havet E et al.** Total knee arthroplasty with the Medial-Pivot knee system: Clinical and radiological outcomes at 9.5 years' mean follow-up. Orthop Traumatol Surg Res 2018; 104: 185–191. doi: 10.1016/J.OTSR.2017.10.016.
41. **Efstathopoulos N, Mavrogenis AF, Lallos S, Nikolaou V, Papagelopoulos PJ, Savvidou OD et al.** 10-year evaluation of the cementless low-contact- stress rotating-platform total knee arthroplasty. J Long Term Eff Med Implants 2009; 19: 255–263. doi: 10.1615/JLONGTERMEFFMEDIMPLANTS.V19.I4.20.
42. **Eggenberger EE, Hurst JM, Morris MJ, Berend KR, Lombardi AV, Crawford DA et al.** Midterm Outcomes of a Bicruciate-Retaining Compared With the Bicruciate-Sacrificing Knee System. J Arthroplasty 2022; 37: S245–S249. doi: 10.1016/J.ARTH.2021.12.010.
43. **Ehrhardt J, Gadinsky N, Lyman S, Markowicz D, Westrich G.** Average 7-year survivorship and clinical results of a newer primary posterior stabilized total knee arthroplasty. HSS J 2011; 7: 120–124. doi: 10.1007/S11420-011-9196-1.
44. **Emerson RH, Higgins LL, Head WC.** The AGC total knee prosthesis at average 11 years. J Arthroplasty 2000; 15: 418–423. doi: 10.1054/ARTH.2000.4337.
45. **Eriksen J, Christensen J, Solgaard S, Schrøder H.** The cementless AGC 2000 knee prosthesis: 20-year results in a consecutive series. Acta Orthop Belg 2009; 75: 225–233. https://europepmc.org/article/med/19492562 [22 Jun. 2023].
46. **Ewald FC, Wright RJ, Poss R, Thomas WH, Mason MD, Sledge CB et al.** Kinematic total knee arthroplasty: a 10- to 14-year prospective follow-up review. J Arthroplasty 1999; 14: 473–480. doi: 10.1016/S0883-5403(99)90104-3.
47. **Faraj AA, Nevelos AB, Nair A.** A 4- to 10-year follow-up study of the Tricon-M noncemented total knee replacement. Orthopedics 2001; 24: 1151–1154. doi: 10.3928/0147-7447-20011201-13.
48. **Faris PM, Keating EM, Farris A, Meding JB, Ritter MA.** Hybrid total knee arthroplasty: 13-year survivorship of AGC total knee systems with average 7 years followup. Clin Orthop Relat Res 2008; 466: 1204–1209. doi: 10.1007/S11999-008-0195-6.
49. **Fernandez-Fairen M, Hernández-Vaquero D, Murcia A, Torres A, Llopis R.** Trabecular metal in total knee arthroplasty associated with higher knee scores: a randomized controlled trial. Clin Orthop Relat Res 2013; 471: 3543–3553. doi: 10.1007/S11999-013-3183-4.
50. **Fetzer GB, Callaghan JJ, Templeton JE, Goetz DD, Sullivan PM, Kelley SS et al**. Posterior cruciate-retaining modular total knee arthroplasty: A 9- to 12-year follow-up investigation. J Arthroplasty 2002; 17: 961–966. doi: 10.1054/arth.2002.34824.
51. **Forster MC, Kothari P, Howard PW.** Minimum 5-year follow-up and radiologic analysis of the all-polyethylene tibial component of the Kinemax Plus system. J Arthroplasty 2002; 17: 196–200. doi: 10.1054/arth.2002.30283.
52. **Garceau SP, Harris NH, Felberbaum DL, Teo GM, Weinblatt AI, Long WJ et al.** Reduced Aseptic Loosening With Fully Cemented Short-Stemmed Tibial Components in Primary Cemented Total Knee Arthroplasty. J Arthroplasty 2020; 35: 1591-1594.e3. doi: 10.1016/J.ARTH.2020.01.084.
53. **Garceau SP, Pivec R, Teo G, Chisari E, Enns PA, Weinblatt AI et al.** Increased Rates of Tibial Aseptic Loosening in Primary Cemented Total Knee Arthroplasty With a Short Native Tibial Stem Design. Journal of the American Academy of Orthopaedic Surgeons 2022; 30: E640–E648. doi: 10.5435/JAAOS-D-21-00536.
54. **Ghalayini SRA, Helm AT, McLauchlan GJ.** Minimum 6 year results of an uncemented trabecular metal tibial component in total knee arthroplasty. Knee 2012; 19: 872–874. doi: 10.1016/J.KNEE.2012.05.001.
55. **Gibon E, Lewallen DG, Larson DR, Stuart MJ, Pagnano MW, Abdel MP et al.** Randomized Clinical Trial of Cementless vs. Cemented Tibial Components: Durable and Reliable at a Mean 10 Years Follow Up. J Arthroplasty 2023; 38: S14–S20. doi: 10.1016/j.arth.2023.03.015.
56. **Gill GS, Joshi AB.** Long-term results of Kinematic Condylar knee replacement. An analysis of 404 knees. J Bone Joint Surg Br 2001; 83: 355–358. doi: 10.1302/0301-620X.83B3.11288.
57. **Gioe TJ, Bowman KR.** A randomized comparison of all-polyethylene and metal-backed tibial components. Clin Orthop Relat Res 2000; 380: 108–115. doi: 10.1097/00003086-200011000-00015.
58. **Gioe TJ, Stroemer ES, Santos ERG.** All-polyethylene and metal-backed tibias have similar outcomes at 10 years: a randomized level I evidence study. Clin Orthop Relat Res 2007; 455: 212–218. doi: 10.1097/01.BLO.0000238863.69486.97.
59. **Giustra F, Bistolfi A, Bosco F, Fresia N, Sabatini L, Berchialla P et al.** Highly cross-linked polyethylene versus conventional polyethylene in primary total knee arthroplasty: comparable clinical and radiological results at a 10-year follow-up. Knee Surg Sports Traumatol Arthrosc 2023; 31: 1082–1088. doi: 10.1007/S00167-022-07226-6.

60. **Glover AW, Santini AJA, Davidson JS, Pope JA.** Mid- to long-term survivorship of oxidised zirconium total knee replacements performed in patients under 50years of age. Knee 2018; 25: 617–622. doi: 10.1016/J.KNEE.2018.03.014.
61. **Goldberg VM, Figgie MP, Figgie HE, Heiple KG, Sobel M.** Use of a total condylar knee prosthesis for treatment of osteoarthritis and rheumatoid arthritis. Long-term results. J Bone Joint Surg Am 1988; 70: 802–811. doi: 10.2106/00004623-198870060-00002.
62. **Goldberg VM, Kraay M.** The outcome of the cementless tibial component: a minimum 14-year clinical evaluation. Clin Orthop Relat Res 2004; 428: 214–220. doi: 10.1097/01.BLO.0000148572.95121.F0.
63. **Grewal R, Rimmer MG, Freeman MAR.** Early migration of prostheses related to long-term survivorship: Comparison of tibial components in knee replacement. Journal of Bone and Joint Surgery - Series B 1992; 74: 239–242. doi: 10.1302/0301-620X.74B2.1544960.
64. **Hack J, Mai S, Siebert W.** Zehn-Jahres-Ergebnisse nach bikondylärem Kniegelenksersatz mit der kreuzbanderhaltenden Knietotalendoprothese NexGen CR. Z Orthop Unfall 2015; 153: 508–515. doi: 10.1055/S-0035-1546040/ID/R088-20/BIB.
65. **Hannon CP, Salih R, Barrack RL, Nunley RM**. Cementless Versus Cemented Total Knee Arthroplasty: Concise Midterm Results of a Prospective Randomized Controlled Trial. J Bone Joint Surg Am 2022; 105: 1430–1434. doi: 10.2106/JBJS.23.00161.
66. **Hamilton DF, Burnett R, Patton JT, Howie CR, Moran M, Simpson A et al**. Implant design influences patient outcome after total knee arthroplasty: a prospective double-blind randomised controlled trial. Bone Joint J 2015; 97-B: 64–70. doi: 10.1302/0301-620X.97B1.34254.
67. **Hamilton DF, Burnett R, Patton JT, MacPherson GJ, Simpson AHRW, Howie CR et al.** Reduction in patient outcomes but implant-derived preservation of function following total knee arthroplasty: longitudinal follow-up of a randomized controlled trial. Bone Joint J 2020; 102-B: 434. doi: 10.1302/0301-620X.102B4.BJJ-2019-0767.R2.
68. **Harwin SF, Kester MA, Malkani AL, Manley MT.** Excellent fixation achieved with cementless posteriorly stabilized total knee arthroplasty. J Arthroplasty 2013; 28: 7–13. doi: 10.1016/J.ARTH.2012.06.006.
69. **Harwin SF, Patel NK, Chughtai M, Khlopas A, Ramkumar PN, Roche M, Mont MA.** Outcomes of Newer Generation Cementless Total Knee Arthroplasty: Beaded Periapatite-Coated vs Highly Porous Titanium-Coated Implants. J Arthroplasty 2017; 32: 2156–2160. doi: 10.1016/J.ARTH.2017.01.044.
70. **Hatayama K, Terauchi M, Saito K, Higuchi H.** Does Residual Varus Alignment Cause Increasing Varus Laxity at a Minimum of Five Years After Total Knee Arthroplasty? J Arthroplasty 2017; 32: 1808–1813, . doi: 10.1016/J.ARTH.2017.01.006.
71. **Hegarty P, Walls A, O'Brien S, Gamble B, Cusick L, Beverland DE et al.** A Prospective Randomized Study Comparing Postoperative Pain, Biological Fixation, and Clinical Outcomes Between Two Uncemented Rotating Platform Tibial Tray Designs. J Arthroplasty 2020; 35: 429–437. doi: 10.1016/J.ARTH.2019.09.029.
72. **Helvie PF, Deckard ER, Meneghini RM.** Cementless Total Knee Arthroplasty Over the Past Decade: Excellent Survivorship in Contemporary Designs. J Arthroplasty 2023; 38. doi: 10.1016/J.ARTH.2023.02.009.
73. **Holland P, Santini AJA, Davidson JS, Pope JA.** Five year survival analysis of an oxidised zirconium total knee arthroplasty. Knee 2013; 20: 384–387. doi: 10.1016/J.KNEE.2012.10.007.
74. **Howell SM, Papadopoulos S, Kuznik K, Ghaly LR, Hull ML.** Does varus alignment adversely affect implant survival and function six years after kinematically aligned total knee arthroplasty? Int Orthop 2015; 39: 2117–2124. doi: 10.1007/S00264-015-2743-5.
75. **Howell SM, Shelton TJ, Hull ML.** Implant Survival and Function Ten Years After Kinematically Aligned Total Knee Arthroplasty. J Arthroplasty 2018; 33: 3678–3684. doi: 10.1016/J.ARTH.2018.07.020.
76. **Hsu RWW, Tsai YH, Huang TJ, Chang JCC.** Hybrid total knee arthroplasty: A 3- to 6-year outcome analysis. Journal of the Formosan Medical Association 1998; 97: 410–415.
77. **Jabbal M, Clement N, Walmsley PJ.** All-polyethylene tibia components have the same functional outcomes and survival, and are more cost-effective than metal-backed components in patients 70 years and older undergoing total knee arthroplasty. Bone Jt Open 2022; 3: 969–976. doi: 10.1302/2633-1462.312.BJO-2022-0063.R1.
78. **Jauregui JJ, Cherian JJ, Pierce TP, Beaver WB, Issa K, Mont MA et al.** Long-Term Survivorship and Clinical Outcomes Following Total Knee Arthroplasty. J Arthroplasty 2015; 30: 2164–2166. doi: 10.1016/j.arth.2015.05.052.
79. **Jeon YS, Shin JS, Jung JH, Kim MK.** Total knee arthroplasty using NexGen LPS-flex improves clinical outcomes without early loosening: Minimum 6-year follow-up results. J Orthop Surg Res 2016; 11: 1–6. doi: 10.1186/s13018-016-0419-5.

80. **Jeon SW, Kim K Il, Song SJ.** Robot-Assisted Total Knee Arthroplasty Does Not Improve Long-Term Clinical and Radiologic Outcomes. J Arthroplasty 2019; 34: 1656–1661. doi: 10.1016/J.ARTH.2019.04.007.
81. **Jiao Y, Zhang X, Askar Mamtimin, Xu B, Li G, Cao L et al.** Comparison of effectiveness of total knee arthroplasty with tantalum monoblock tibial component and cemented tibial plateau prosthesis in patients of different ages. Chinese Journal of Reparative and Reconstructive Surgery 2021; 35: 1563–1573. doi: 10.7507/1002-1892.202104007.
82. **Kaipel M, Klikovics J, Sinz G, Szabo S, Weinoehrl S, Hausner T et al.** Convincing long-term results independent from the postoperative leg alignment following cementless total knee arthroplasty. Knee 2020; 27: 552–557. doi: 10.1016/J.KNEE.2019.11.016.
83. **Kajetanek C, Bouyer B, Ollivier M, Boisrenoult P, Pujol N, Beaufils P et al.** Mid-term survivorship of Mini-keel versus Standard keel in total knee replacements: Differences in the rate of revision for aseptic loosening. Orthop Traumatol Surg Res 2016; 102: 611–617. doi: 10.1016/J.OTSR.2016.05.007.
84. **Kamath AF, Lee GC, Sheth NP, Nelson CL, Garino JP, Israelite CL et al**. Prospective Results of Uncemented Tantalum Monoblock Tibia in Total Knee Arthroplasty: Minimum 5-Year Follow-up in Patients Younger Than 55 Years. J Arthroplasty 2011; 26: 1390–1395. doi: 10.1016/J.ARTH.2011.06.030.
85. **Karachalios T, Roidis N, Giotikas D, Bargiotas K, Varitimidis S, Malizos KN et al.** A mid-term clinical outcome study of the Advance Medial Pivot knee arthroplasty. Knee 2009; 16: 484–488. doi: 10.1016/J.KNEE.2009.03.002.
86. **Karachalios T, Varitimidis S, Bargiotas K, Hantes M, Roidis N, Malizos KN et al.** An 11- to 15-year clinical outcome study of the Advance Medial Pivot total knee arthroplasty: Pivot knee arthroplasty. Bone and Joint Journal 2016; 98-B: 1050–1055. doi: 10.1302/0301-620X.98B8.36208.
87. **Khaw FM, Kirk LMG, Gregg PJ**. Survival analysis of cemented Press-Fit Condylar total knee arthroplasty. J Arthroplasty 2001; 16: 161–167. doi: 10.1054/ARTH.2001.20254.
88. **Khaw FM, Surgery O, Kirk LMG, Morris RW, Gregg PJ.** A randomised, controlled trial of cemented versus cementless press-fit condylar total knee replacement: TEN-YEAR SURVIVAL ANALYSIS. J Bone Joint Surg Br 2002; 84-B: 658–666. doi: 10.1302/0301-620X.84B5.0840658.
89. **Kim GW, Jin QH, Lim JH, Song EK, Seon JK.** No difference of survival between cruciate retaining and substitution designs in high flexion total knee arthroplasty. Scientific reports 2021; 11(1): 6537. doi:10.1038/s41598-021-85892-1
90. **Kim YH, Yoon SH, Kim JS.** The long-term results of simultaneous fixed-bearing and mobile-bearing total knee replacements performed in the same patient. J Bone Joint Surg Br 2007; 89: 1317–1323. doi: 10.1302/0301-620X.89B10.19223.
91. **Kim YH, Park JW, Kim JS.** Computer-navigated versus conventional total knee arthroplasty a prospective randomized trial. J Bone Joint Surg Am 2012; 94: 2017–2024. doi: 10.2106/JBJS.L.00142.
92. **Kim YH, Park JW, Kim JS.** High-flexion total knee arthroplasty: survivorship and prevalence of osteolysis: results after a minimum of ten years of follow-up. J Bone Joint Surg Am 2012; 94: 1378–1384. doi: 10.2106/JBJS.K.01229.
93. **Kim YH, Park JW, Lim HM, Park ES.** Cementless and cemented total knee arthroplasty in patients younger than fifty five years. Which is better? Int Orthop 2014; 38: 297–303. doi: 10.1007/S00264-013-2243-4.
94. **Kim YH, Park JW.** Comparison of highly cross-linked and conventional polyethylene in posterior cruciate-substituting total knee arthroplasty in the same patients. J Bone Joint Surg Am 2014; 96: 1807–1813. doi: 10.2106/JBJS.M.01605.
95. **Kim YH, Park JW, Kim JS, Kulkarni SS, Kim YH.** Long-term clinical outcomes and survivorship of press-fit condylar sigma fixed-bearing and mobile-bearing total knee prostheses in the same patients. J. Bone Joint Surg. Am 2014; 96: e168. doi: 10.2106/JBJS.M.01130.
96. **Kim YH, Park JW, Kim JS, Lee JH**. Highly Crosslinked-remelted versus Less-crosslinked Polyethylene in Posterior Cruciate-retaining TKAs in the Same Patients. Clin Orthop Relat Res 2015; 473: 3588–3594. doi: 10.1007/S11999-015-4425-4.
97. **Kim YH, Park JW, Kim JS.** Clinical Outcome of Medial Pivot Compared With Press-Fit Condylar Sigma Cruciate-Retaining Mobile-Bearing Total Knee Arthroplasty. J Arthroplasty 2017; 32: 3016–3023. doi: 10.1016/J.ARTH.2017.05.022.
98. **Kim YH, Park JW, Kim JS.** The Clinical Outcome of Computer-Navigated Compared with Conventional Knee Arthroplasty in the Same Patients: A Prospective, Randomized, Double-Blind, Long-Term Study. J Bone Joint Surg Am 2017; 99: 989–996. doi: 10.2106/JBJS.16.00791.
99. **Kim YH, Park JW, Kim JS.** Does Computer Navigation in Knee Arthroplasty Improve Functional Outcomes in Young Patients? A Randomized Study. Clin Orthop Relat Res 2018; 476: 6–15. doi: 10.1007/S11999.0000000000000000.

100. **Kim YH, Park JW.** Long-Term Assessment of Highly Cross-Linked and Compression-Molded Polyethylene Inserts for Posterior Cruciate-Substituting TKA in Young Patients: A Concise Follow-up of a Previous Report. J Bone Joint Surg Am 2020; 102: 1623–1627. doi: 10.2106/JBJS.20.00261.
101. **Kim YH, Park JW, Jang YS.** Long-Term (Up to 27 Years) Prospective, Randomized Study of Mobile-Bearing and Fixed-Bearing Total Knee Arthroplasties in Patients. J Arthroplasty 2021; 36: 1330–1335. doi: 10.1016/J.ARTH.2020.10.050.
102. **Kim YH, Park JW, Jang YS.** The 22 to 25-Year Survival of Cemented and Cementless Total Knee Arthroplasty in Young Patients. J Arthroplasty 2021; 36: 566–572. doi: 10.1016/J.ARTH.2020.08.001.
103. **Kim YH, Park JW, Jang YS.** 20-Year Minimum Outcomes and Survival Rate of High-Flexion Versus Standard Total Knee Arthroplasty. J Arthroplasty 2021; 36: 560–565. doi: 10.1016/J.ARTH.2020.07.084.
104. **Knight JL, Atwater RD, Grothaus L.** Clinical results of the modular porous-coated anatomic (PCA) total knee arthroplasty with cement: a 5-year prospective study. Orthopedics 1997; 20: 1025–1033. doi: 10.3928/0147-7447-19971101-08.
105. **Kwong LM, Nielsen ES, Ruiz DR, Hsu AH, Dines MD, Mellano CM.** Cementless total knee replacement fixation: A contemporary durable solution-affirms. BJJ 2014; 96B: 87–92. doi: 10.1302/0301-620X.96B11.34327.
106. **Lachiewicz PF, Soileau ES.** Patella maltracking in posterior-stabilized total knee arthroplasty. Clin Orthop Relat Res 2006; 452: 155–158. doi: 10.1097/01.BLO.0000238803.97713.7D.
107. **Lachiewicz PF, Soileau ES.** Fixation, survival and osteolysis with a modern posterior-stabilized total knee arthroplasty. J Arthroplasty 2014; 29: 66–70. doi: 10.1016/J.ARTH.2013.05.002.
108. **Laskin RS.** Tricon-M uncemented total knee arthroplasty. A review of 96 knees followed for longer than 2 years. J Arthroplasty 1988; 3: 27–38. doi: 10.1016/S0883-5403(88)80050-0.
109. **Law JI, Hurst JM, Morris MJ, Berend KR, Lombardi A V., Crawford DA et al.** Midterm Outcomes and Survivorship of Anterior Stabilized Versus Cruciate Retaining Bearing in Primary Total Knee Arthroplasty. J Arthroplasty 2021; 36: 998–1002. doi: 10.1016/J.ARTH.2020.09.053.
110. **Lee BS, Chung JW, Kim JM, Kim KA, Bin S Il.** High-flexion prosthesis improves function of TKA in Asian patients without decreasing early survivorship. Clin Orthop Relat Res 2013; 471: 1504–1511. doi: 10.1007/S11999-012-2661-4.
111. **Lee BS, Lee SJ, Kim JM, Lee DH, Cha EJ, Bin S et al.** No impact of severe varus deformity on clinical outcome after posterior stabilized total knee arthroplasty. Knee Surg Sports Traumatol Arthrosc 2011; 19: 960–966. doi: 10.1007/S00167-010-1316-9.
112. **Lee WG, Song EK, Choi SW, Jin QH, Seon JK.** Comparison of Posterior Cruciate-Retaining and High-Flexion Cruciate-Retaining Total Knee Arthroplasty Design. J Arthroplasty 2020; 35: 752–755. doi: 10.1016/J.ARTH.2019.10.008.
113. **Lombardi A V., Mallory TH, Fada RA, Hartman JF, Capps SG, Kefauver CA et al.** An algorithm for the posterior cruciate ligament in total knee arthroplasty. Clin Orthop Relat Res 2001; 392: 75–87. doi: 10.1097/00003086-200111000-00010.
114. **Louwerens JKG, Hockers N, Achten G, Sierevelt IN, Nolte PA, van Hove RP et al.** No clinical difference between TiN-coated versus uncoated cementless CoCrMo mobile-bearing total knee arthroplasty; 10-year follow-up of a randomized controlled trial. Knee Surg Sports Traumatol Arthrosc 2021; 29: 750–756. doi: 10.1007/S00167-020-05997-4.
115. **Lunebourg A, Parratte S, Gay A, Ollivier M, Garcia-Parra K, Argenson JN et al.** Lower function, quality of life, and survival rate after total knee arthroplasty for posttraumatic arthritis than for primary arthritis. Acta Orthop 2015; 86: 189. doi: 10.3109/17453674.2014.979723.
116. **Malin AS, Callaghan JJ, Bozic KJ, Liu SS, Goetz DD, Sullivan N et al.** Routine Surveillance of Modular PFC TKA Shows Increasing Failures after 10 Years. Clin Orthop Relat Res 2010; 468: 2469. doi: 10.1007/S11999-010-1303-Y.
117. **Mayman D, Bourne RB, Rorabeck CH, Vaz M, Kramer J**. Resurfacing versus not resurfacing the patella in total knee arthroplasty: 8- to 10-year results. J Arthroplasty 2003; 18: 541–545. doi: 10.1016/S0883-5403(03)00150-5.
118. **McCalden RW, Hart GP, MacDonald SJ, Naudie DD, Howard JH, Bourne RB et al.** Clinical Results and Survivorship of the GENESIS II Total Knee Arthroplasty at a Minimum of 15 Years. J Arthroplasty 2017; 32: 2161–2166, . doi: 10.1016/J.ARTH.2017.02.006.
119. **McMahon SE, Doran E, O'Brien S, Cassidy RS, Boldt JG, Beverland DE et al.** Seventeen to Twenty Years of Follow-Up of the Low Contact Stress Rotating-Platform Total Knee Arthroplasty With a Cementless Tibia in All Cases. J Arthroplasty 2019; 34: 508–512. doi: 10.1016/J.ARTH.2018.11.024.

120. **Meding JB, Ritter MA, Faris PM.** Total knee arthroplasty with 4.4 mm of tibial polyethylene: 10-year followup. Clin Orthop Relat Res 2001; 388: 112–117. doi: 10.1097/00003086-200107000-00017.
121. **Meding JB, Wing JT, Ritter MA.** Total knee arthroplasty with 4.4 mm of tibial polyethylene an update. J Arthroplasty 2010; 25: 772–774. doi: 10.1016/J.ARTH.2009.05.026.
122. **Meena A, Hoser C, Abermann E, Hepperger C, Raj A, Fink C et al.** Total knee arthroplasty improves sports activity and the patient-reported functional outcome at mid-term follow-up. Knee Surg Sports Traumatol Arthrosc 2023; 31: 905–913. doi: 10.1007/S00167-022-07025-Z.
123. **Mohammed A, Metcalfe A, Woodnutt D.** Medium term outcome of Titanium Nitride, mobile bearing total knee Replacement. Acta Orthop Belg 2014; 80: 269–275.
124. **Mont MA, Yoon TR, Krackow KA, Hungerford DS.** Eliminating patellofemoral complications in total knee arthroplasty: Clinical and radiographic results of 121 consecutive cases using the Duracon system. J Arthroplasty 1999; 14: 446–455. doi: 10.1016/S0883-5403(99)90100-6.
125. **Moran CG, Pinder IM, Lees TA, Midwinter MJ.** Survivorship analysis of the uncemented porous-coated anatomic knee replacement. J Bone Joint Surg Am 1991; 73: 848–857. doi: 10.2106/00004623-199173060-00007.
126. **Nam D, Lawrie CM, Salih R, Nahhas CR, Barrack RL, Nunley RM et al.** Cemented Versus Cementless Total Knee Arthroplasty of the Same Modern Design: A Prospective, Randomized Trial. J Bone Joint Surg Am 2019; 101: 1185–1192. doi: 10.2106/JBJS.18.01162.
127. **Napier RJ, O'Neill C, O'Brien S, Doran E, Mockford B, Boldt J et al.** A prospective evaluation of a largely cementless total knee arthroplasty cohort without patellar resurfacing: 10-year outcomes and survivorship. BMC Musculoskelet Disord 2018; 19. doi: 10.1186/S12891-018-2128-1.
128. **Oliver WM, Arthur CHC, Wood AM, Clayton RAE, Brenkel IJ, Walmsley P et al.** Excellent Survival and Good Outcomes at 15 Years Using the Press-Fit Condylar Sigma Total Knee Arthroplasty. J Arthroplasty 2018; 33: 2524–2529. doi: 10.1016/J.ARTH.2018.03.048.
129. **Ollivier M, Parratte S, Lino L, Flecher X, Pesenti S, Argenson JN et al.** No Benefit of Computer-assisted TKA: 10-year Results of a Prospective Randomized Study. Clin Orthop Relat Res 2018; 476: 126. doi: 10.1007/S11999.0000000000000021.
130. **Parsch D, Krüger M, Moser MT, Geiger F.** Follow-up of 11-16 years after modular fixed-bearing TKA. Int Orthop 2009; 33: 431–435. doi: 10.1007/S00264-008-0543-X.
131. **Patil SS, Branovacki G, Martin MR, Pulido PA, Levy YD, Colwell CW et al**. 14-year median follow-up using the press-fit condylar sigma design for total knee arthroplasty. J Arthroplasty 2013; 28: 1286–1290. doi: 10.1016/J.ARTH.2012.11.014.
132. **Pelt CE, Gililland JM, Doble J, Stronach BM, Peters CL.** Hybrid total knee arthroplasty revisited: midterm followup of hybrid versus cemented fixation in total knee arthroplasty. Biomed Res Int 2013; 2013:854871. doi: 10.1155/2013/854871.
133. **Powell AJ, Crua E, Chong BC, Gordon R, McAuslan A, Pitto RP et al.** A randomized prospective study comparing mobile-bearing against fixed-bearing PFC Sigma cruciate-retaining total knee arthroplasties with ten-year minimum follow-up. Bone Joint J 2018; 100-B: 1336–1344. doi: 10.1302/0301-620X.100B10.BJJ-2017-1450.R1.
134. **Pulido L, Abdel MP, Lewallen DG, Stuart MJ, Sanchez-Sotelo J, Hanssen AD et al.** Trabecular metal tibial components were durable and reliable in primary total knee arthroplasty: a randomized clinical trial. Clin Orthop Relat Res 2015; 473: 34–42. doi: 10.1007/S11999-014-3585-Y.
135. **Rajgopal A, Kumar S, Aggarwal K.** Evaluating Long Term Outcomes and Survivorship of Cruciate Retaining and Sacrificing Knee Replacements Done for Degenerative Arthritis in Patients Under 55 Years. Indian J Orthop 2021; 55: 1180–1185. doi: 10.1007/S43465-021-00460-Z.
136. **Ranawat CS, Flynn WF, Saddler S, Hansraj KK, Maynard MJ.** Long-term results of the total condylar knee arthroplasty; A 15-year survivorship study. Clin Orthop Relat Res 1993; (286), 94–102.
137. **Ranawat CS, Flynn WF, Deshmukh RG.** Impact of modern technique on long-term results of total condylar knee arthroplasty. Clin Orthop Relat Res 1994; 309: 131–135. doi: 10.1016/0883-5403(94)90183-x.
138. **Rassir R, Puijk R, Singh J, Sierevelt IN, Vergroesen DA, Nolte PA et al.** Long-Term Clinical Performance of an Uncemented, Mobile Bearing, Anterior Stabilized Knee System and the Impact of Previous Knee Surgery. J Arthroplasty 2022; 37: 2041–2048. doi: 10.1016/J.ARTH.2022.04.039.
139. **Restrepo S, Smith EB, Hozack WJ.** Excellent mid-term follow-up for a new 3D-printed cementless total knee arthroplasty. Bone Joint J 2021; 103-B: 32–37. doi: 10.1302/0301-620X.103B6.BJJ-2020-2096.R1.
140. **Rhee SJ, Hong SM, Suh JT.** High-Flexion Total Knee Arthroplasty Using NexGen LPS-Flex System: Minimum 5-year Follow-up Results. Knee Surg Relat Res 2015; 27: 156–162. doi: 10.5792/KSRR.2015.27.3.156.

141. **Riaz O, Aqil A, Sisodia G, Chakrabarty G.** P.F.C Sigma cruciate retaining fixed-bearing versus mobile-bearing knee arthroplasty: a prospective comparative study with minimum 10-year follow-up. Eur J Orthop Surg Traumatol 2017; 27: 1145–1149. doi: 10.1007/S00590-017-1920-1.
142. **Rinonapoli E, Mancini GB, Azzara A, Aglietti P.** Long-term results and survivorship analysis of 89 total condylar knee prostheses. J Arthroplasty 1992; 7: 241–246. doi: 10.1016/0883-5403(92)90043-P.
143. **Ritter MA, Wing JT, Berend ME, Davis KE, Meding JB.** The clinical effect of gender on outcome of total knee arthroplasty. J Arthroplasty 2008; 23: 331–336. doi: 10.1016/J.ARTH.2007.10.031.
144. **Ritter MA, Meneghini RM.** Twenty-Year Survivorship of Cementless Anatomic Graduated Component Total Knee Arthroplasty. J Arthroplasty 2010; 25: 507–513. doi: 10.1016/J.ARTH.2009.04.018.
145. **Ritter MA, Keating EM, Sueyoshi T, Davis KE, Barrington JW, Emerson RH et al.** Twenty-Five-Years and Greater, Results After Nonmodular Cemented Total Knee Arthroplasty. J Arthroplasty 2016; 31: 2199–2202. doi: 10.1016/J.ARTH.2016.01.043.
146. **Robertsson O, Scott G, Freeman MAR.** Ten-year survival of the cemented Freeman-Samuelson primary knee arthroplasty. Data from the Swedish Knee Arthroplasty Register and the Royal London Hospital. J Bone Joint Surg Br 2000; 82: 506–507. doi: 10.1302/0301-620X.82B4.10342.
147. **Rodricks DJ, Patil S, Pulido P, Colwell CW.** Press-fit condylar design total knee arthroplasty. Fourteen to seventeen-year follow-up. J Bone Joint Surg Am 2007; 89: 89–95. doi: 10.2106/JBJS.E.00492.
148. **Rossi SMP, Perticarini L, Mosconi M, Ghiara M, Benazzo F.** Ten-year outcomes of a nitrided Ti-6Al-4V titanium alloy fixed-bearing total knee replacement with a highly crosslinked polyethylene-bearing in patients with metal allergy. Knee 2020; 27: 1519–1524. doi: 10.1016/j.knee.2020.08.007.
149. **Samuelson K, Nelson L.** An all-polyethylene cementless tibial component. A five- to nine-year follow-up study. Clin Orthop Relat Res 1990; 260: 93–97. doi: 10.1097/00003086-199011000-00017.
150. **Sando T, McCalden RW, Bourne RB, MacDonald SJ, Somerville LE.** Ten-year results comparing posterior cruciate-retaining versus posterior cruciate-substituting total knee arthroplasty. J Arthroplasty 2015; 30: 210–215. doi: 10.1016/J.ARTH.2014.09.009.
151. **Santini AJA, Raut V.** Ten-year survival analysis of the PFC total knee arthroplasty-a surgeon's first 99 replacements. Int Orthop 2008; 32: 459–465. doi: 10.1007/S00264-007-0351-8.
152. **Schiavone Panni A, Falez F, D'Apolito R, Corona K, Perisano C, Vasso M et al.** Long-term follow-up of a non-randomised prospective cohort of one hundred and ninety two total knee arthroplasties using the NexGen implant. Int Orthop 2017; 41: 1155–1162. doi: 10.1007/S00264-017-3438-X.
153. **Schotanus MGM, Boonen B, van der Weegen W, Hoekstra H, van Drumpt R, Borghans R et al.** No difference in mid-term survival and clinical outcome between patient-specific and conventional instrumented total knee arthroplasty: a randomized controlled trial. Knee Surg Sports Traumatol Arthrosc 2019; 27: 1463–1468. doi: 10.1007/S00167-018-4968-5.
154. **Schrøder HM, Berthelsen A, Hassani G, Hansen EB, Solgaard S.** Cementless porous-coated total knee arthroplasty: 10-year results in a consecutive series. J Arthroplasty 2001; 16: 559–567. doi: 10.1054/ARTH.2001.23565.
155. **Scott RD.** The incidence and causes of re-operation after press-fit condylar (PFC) total knee arthroplasty. Journal of Orthopaedic Science 1997; 2: 46–52. doi: 10.1007/BF01239758.
156. **Scott CEH, Clement ND, MacDonald DJ, Hamilton DF, Gaston P, Howie CR et al.** Five-year survivorship and patient-reported outcome of the Triathlon single-radius total knee arthroplasty. Knee Surg Sports Traumatol Arthrosc 2015; 23: 1676–1683. doi: 10.1007/S00167-014-2922-8.
157. **Scott CEH, Bell KR, Ng RT, MacDonald DJ, Patton JT, Burnett R et al.** Excellent 10-year patient-reported outcomes and survival in a single-radius, cruciate-retaining total knee arthroplasty. Knee Surg Sports Traumatol Arthrosc 2019; 27: 1106–1115. doi: 10.1007/S00167-018-5179-9.
158. **Selvan DR, Santini AJA, Davidson JS, Pope JA.** The Medium-Term Survival Analysis of an All-Polyethylene Tibia in a Single-Series Cohort of Over 1000 Knees. J Arthroplasty 2020; 35: 2837–2842. doi: 10.1016/J.ARTH.2020.05.017.
159. **Seon JK, Park SJ, Lee KB, Yoon TR, Kozanek M, Song EK et al.** Range of motion in total knee arthroplasty: A prospective comparison of high-flexion and standard cruciate-retaining designs. J. Bone Jt. Surg 2009; 91: 672–679. doi: 10.2106/JBJS.H.00300.
160. **Sextro GS, Berry DJ, Rand JA.** Total knee arthroplasty using cruciate-retaining kinematic condylar prosthesis. Clin Orthop Relat Res 2001; 388: 33–40. doi: 10.1097/00003086-200107000-00007.
161. **Sheridan GA, Cassidy RS, McKee C, Hughes I, Hill JC, Beverland DE et al.** Survivorship of 500 Cementless Total Knee Arthroplasties in Patients Under 55 Years of Age. J Arthroplasty 2023; 38(5), 820–823. doi: 10.1016/J.ARTH.2022.10.035.

162. **Shi W, Jiang Y, Wang C, Zhang H, Wang Y, Li T.** Comparative study on mid- And long-term clinical effects of medial pivot prosthesis and posterior-stabilized prosthesis after total knee arthroplasty. J Orthop Surg Res 2020; 15: 1–9. doi: 10.1186/S13018-020-01951-9/FIGURES/5.
163. **Smith AJ, Wood DJ, Li MG.** Total knee replacement with and without patellar resurfacing: a prospective, randomised trial using the profix total knee system. J Bone Joint Surg Br 2008; 90: 43–49. doi: 10.1302/0301-620X.90B1.18986.
164. **Song SJ, Lee HW, Bae DK, Park CH.** High Incidence of Tibial Component Loosening After Total Knee Arthroplasty Using Ceramic Titanium-Nitride-Coated Mobile Bearing Prosthesis in Moderate to Severe Varus Deformity: A Matched-Pair Study Between Ceramic-Coated Mobile Bearing and Fixed Bearing Prostheses. J Arthroplasty 2020; 35: 1003–1008. doi: 10.1016/J.ARTH.2019.11.034.
165. **Stempin R, Stempin K, Kaczmarek W, Dutka J.** Midterm Results of Cementless Total Knee Arthroplasty: A Retrospective Case Series. Open Orthop J 2018; 12: 196–202. doi: 10.2174/1874325001812010196.
166. **Stevenson KL, Blackburn BE, Da Silva AZ, Erickson JA, Anderson LA, Pelt CE et al.** High Survivorship of a Modular Titanium Baseplate Independent of Body Mass Index and Malalignment. J Arthroplasty 2022; 37: S216–S220. doi: 10.1016/J.ARTH.2022.02.006.
167. **Stukenborg-Colsman C, Wirth CJ.** Knee endoprosthesis and clinical aspects. Orthopade 2000; 29: 732–738. doi: 10.1007/S001320050519.
168. **Tanavalee A, Ngarmukos S, Tantavisut S, Limtrakul A.** High-flexion TKA in patients with a minimum of 120 degrees of pre-operative knee flexion: outcomes at six years of follow-up. Int Orthop 2011; 35: 1321. doi: 10.1007/S00264-010-1140-3.
169. **Tarazi JM, Salem HS, Ehiorobo JO, Sodhi N, Mont MA, Harwin SF et al.** Cementless Tritanium Baseplate Total Knee Arthroplasty: Survivorship and Outcomes at 5-Year Minimum Follow-Up. J Knee Surg 2020; 33: 862–865. doi: 10.1055/S-0040-1712983.
170. **Tibbo ME, Limberg AK, Perry KI, Pagnano MW, Stuart MJ, Hanssen AD et al.** Effect of Coronal Alignment on 10-Year Survivorship of a Single Contemporary Total Knee Arthroplasty. J Clin Med 2021; 10: 1–9. doi: 10.3390/JCM10010142.
171. **Toyoda S, Kaneko T, Mochizuki Y, Hada M, Takada K, Ikegami H et al.** Minimally invasive surgery total knee arthroplasty is less popular, but the prosthesis designed specifically for MIS provides good survival and PROMs with a minimum follow-up of 10 years. J Orthop Surg Res 2021; 16: 1–9. doi: 10.1186/S13018-021-02254-3.
172. **Tucker A, O’Brien S, Doran E, Gallagher N, Beverland DE.** Total Knee Arthroplasty in Severe Valgus Deformity Using a Modified Technique—A 10-Year Follow-Up Study. J Arthroplasty 2019; 34: 40-46.e1. doi: 10.1016/j.arth.2018.09.002.
173. **Ueyama H, Kanemoto N, Minoda Y, Yamamoto N, Taniguchi Y, Nakamura H et al.** Long-term clinical outcomes of medial pivot total knee arthroplasty for Asian patients: A mean 10-year follow-up study. Knee 2020; 27: 1778–1786. doi: 10.1016/J.KNEE.2020.09.009.
174. **van Hove RP, Brohet RM, van Royen BJ, Nolte PA.** No clinical benefit of titanium nitride coating in cementless mobile-bearing total knee arthroplasty. Knee Surg Sports Traumatol Arthrosc 2015; 23: 1833–1840. doi: 10.1007/S00167-014-3359-9.
175. **Verburg H, Mathijssen NMC, Niesten DD, Verhaar JAN, Pilot P.** Comparison of Mini-Midvastus and Conventional Total Knee Arthroplasty with Clinical and Radiographic Evaluation: A Prospective Randomized Clinical Trial with 5-Year Follow-up. J Bone Joint Surg Am 2016; 98: 1014–1022. doi: 10.2106/JBJS.15.00654.
176. **Vessely MB, Whaley AL, Harmsen WS, Schleck CD, Berry DJ.** The Chitranjan Ranawat Award: Long-term survivorship and failure modes of 1000 cemented condylar total knee arthroplasties. Clin Orthop Relat Res 2006; 452: 28–34. doi: 10.1097/01.BLO.0000229356.81749.11.
177. **Weir DJ, Moran CG, Pinder IM.** Kinematic condylar total knee arthroplasty. 14-year survivorship analysis of 208 consecutive cases. J Bone Joint Surg Br 1996; 78: 907–911. doi: 10.1302/0301-620X78B6.6678.
178. **Worland RL, Johnson G, Alemparte J, Jessup DE, Keenan J, Norambuena N et al.** Ten to fourteen year survival and functional analysis of the AGC total knee replacement system. Knee 2002; 9: 133–137. doi: 10.1016/S0968-0160(01)00146-6.
179. **Wright RJ, Ewald FC, Thomas WH, Mason MD, Poss R, Sledge CB et al.** Total knee arthroplasty with the kinematic prosthesis. Results after five to nine years: a follow-up note - PubMed [Online]. J Bone Joint Surg Am 1990; 72: 1003–1009.
180. **Yang HY, Seon JK, Shin YJ, Lim HA, Song EK.** Robotic Total Knee Arthroplasty with a Cruciate-Retaining Implant: A 10-Year Follow-up Study. Clin Orthop Surg 2017; 9: 169. doi: 10.4055/CIOS.2017.9.2.169.

181. **Yang J, Nahhas CR, Salzano MB, Ruzich GP, Jacobs JJ, Paprosky WG et al.** Early outcomes of a modern cemented total knee arthroplasty : is tibial loosening a concern? Bone Joint J 2021; 103-B: 51–58. doi: 10.1302/0301-620X.103B6.BJJ-2020-1972.R1.
182. **Youm YS, Cho S Do, Lee SH, Cho HY.** Total Knee Arthroplasty Using a Posterior Cruciate Ligament Sacrificing Medial Pivot Knee: Minimum 5-year Follow-up Results. Knee Surg Relat Res 2014; 26: 135–140. doi: 10.5792/KSRR.2014.26.3.135.
183. **Young SW, Walker ML, Bayan A, Briant-Evans T, Pavlou P, Farrington B et al.** No Difference in 2-year Functional Outcomes Using Kinematic versus Mechanical Alignment in TKA: A Randomized Controlled Clinical Trial. Clin Orthop Relat Res 2017; 475: 9–20. doi: 10.1007/S11999-016-4844-X.
184. **Young SW, Sullivan NPT, Walker ML, Holland S, Bayan A, Farrington B et al.** No Difference in 5-year Clinical or Radiographic Outcomes Between Kinematic and Mechanical Alignment in TKA: A Randomized Controlled Trial. Clin Orthop Relat Res 2020; 478: 1271–1279. doi: 10.1097/CORR.0000000000001150.
185. **Zaki SH, Rafiq I, Kapoor A, Raut V, Gambhir AK, Porter ML et al.** Medium-term results with the Press Fit Condylar (PFC) Sigma knee prosthesis the Wrightington experience. Acta Orthop Belg 2007; 73: 55–59.
186. **Zhu M, Ang CL, Yeo SJ, Lo NN, Chia SL, Chong HC et al.** Minimally Invasive Computer-Assisted Total Knee Arthroplasty Compared With Conventional Total Knee Arthroplasty: A Prospective 9-Year Follow-Up. J Arthroplasty 2016; 31: 1000–1004. doi: 10.1016/J.ARTH.2015.11.023.

References meta-analysis RSA studies.

1. **Adalberth G, Nilsson KG, Byström S, Kolstad K, Mallmin H, Milbrink J et al.** Stability assessment of a moderately conforming all-polyethylene tibial component in total knee arthroplasty: a prospective RSA study with 2 years of follow-up of the Kinemax Plus design. Am J Knee Surg 1999; 12: 233–240.
2. **Adalberth G, Nilsson KG, Byström S, Kolstad K, Milbrink J.** All-polyethylene versus metal-backed and stemmed tibial components in cemented total knee arthroplasty. A prospective, randomised RSA study. J Bone Joint Surg Br 2001; 83: 825–831. doi: 10.1302/0301-620x.83b6.11430.
3. **Adalberth G, Nilsson KG, Byström S, Kolstad K, Milbrink J.** Low-conforming all-polyethylene tibial component not inferior to metal-backed component in cemented total knee arthroplasty: prospective, randomized radiostereometric analysis study of the AGC total knee prosthesis. J Arthroplasty 2000; 15: 783–792. doi: 10.1054/arth.2000.8101.
4. **Adalberth G, Nilsson KG, Kärrholm J, Hassander H.** Fixation of the tibial component using CMW-1 or Palacos bone cement with gentamicin: similar outcome in a randomized radiostereometric study of 51 total knee arthroplasties. Acta Orthop 2002; 73: 531–538. doi: 10.1080/000164702321022802.
5. **Albrektsson BE, Carlsson L V, Freeman MA, Herberts P, Ryd L.** Proximally cemented versus uncemented Freeman-Samuelson knee arthroplasty. A prospective randomised study. J Bone Joint Surg Br 1992; 74-B: 233–238. doi: 10.1302/0301-620X.74B2.1544959.
6. **Albrektsson BE, Ryd L, Carlsson L V, Freeman MA, Herberts P, Regnér L et al.** The effect of a stem on the tibial component of knee arthroplasty. A roentgen stereophotogrammetric study of uncemented tibial components in the Freeman-Samuelson knee arthroplasty. J Bone Joint Surg Br 1990; 72: 252–258. doi: 10.1302/0301-620x.72b2.2312565.
7. **Andersen MR, Winther N, Lind T, Schrøder H, Flivik G, Petersen MM et al.** Monoblock versus modular polyethylene insert in uncemented total knee arthroplasty: A randomized RSA study with 2-year follow-up of 53 patients. Acta Orthop 2016; 87: 607–614. doi: 10.1080/17453674.2016.1233654.
8. **Catani F, Leardini A, Ensini A, Cucca G, Bragonzoni L, Toksvig-Larsen S et al.** The stability of the cemented tibial component of total knee arthroplasty: posterior cruciate-retaining versus posterior-stabilized design. J Arthroplasty 2004; 19: 775–782. doi: 10.1016/j.arth.2004.01.013.
9. **Christensson, A; Tveit, M, Kesteris, U, Flivik G.** Similar migration for medial congruent and cruciate-retaining tibial components in an anatomic TKA system: a randomized controlled trial of 60 patients followed with RSA for 2 years. Acta Orthop 2022; 93: 68–74. doi: 10.1080/17453674.2021.1983709.
10. **Dalén T, Nilsson KG**. VersaBond bone cement prospective randomized study of the clinical properties of a new bone cement in total knee replacement. Knee 2005; 12: 311–317. doi: 10.1016/J.KNEE.2004.11.004.
11. **Dunbar MJ, Laende EK, Collopy D, Richardson CG.** Stable migration of peri-apatite-coated uncemented tibial components in a multicentre study. Bone and Joint Journal 2017; 99: 1596–1602. doi: 10.1302/0301-620X.99B12.BJJ-2016-1118.R2.
12. **Dunbar MJ, Wilson DA, Hennigar AW, Amirault JD, Gross M, Reardon GP et al.** Fixation of a trabecular metal knee arthroplasty component. A prospective randomized study. J Bone Joint Surg Am 2009; 91: 1578–1586. doi: 10.2106/jbjs.h.00282.
13. **Ejaz A, Laursen AC, Jakobsen T, Rasmussen S, Nielsen PT, Laursen MB.** Absence of a Tourniquet Does Not Affect Fixation of Cemented TKA: A Randomized RSA Study of 70 Patients. J Arthroplasty 2005; 30: 2128–2132. doi: 10.1016/j.arth.2015.05.058.
14. **Hansson U, Ryd L, Toksvig-Larsen S**. A randomised RSA study of Peri-Apatite HA coating of a total knee prosthesis. Knee 2008; 15: 211–216. doi: 10.1016/j.knee.2008.01.013.
15. **Hasan S, Hamersveld KTV, Van de Mheen PJM, Kaptein BL, Nelissen RGHH, Toksvig-Larsen S.** Migration of a novel 3D-printed cementless versus a cemented total knee arthroplasty: Two-year results of a randomized controlled trial using radiostereometric analysis. BJJ 2020; 102: 1016–1024. doi: 10.1302/0301-620X.102B8.BJJ-2020-0054.R1.
16. **Hasan S, Marang-Van De Mheen P, Kaptein BL, Nelissen RGHH, Toksvig-Larsen S.** All-polyethylene versus metal-backed posterior stabilized total knee arthroplasty: similar 2-year results of a randomized radiostereometric analysis study. Acta Orthop 2019; 90: 590–595. doi: 10.1080/17453674.2019.1668602.
17. **Henricson A, Dalén T, Nilsson KG**. Mobile bearings do not improve fixation in cemented total knee arthroplasty. Clin Orthop Relat Res 2006; 448: 114–121. doi: 10.1097/01.blo.0000224004.40883.ab.
18. **Henricson A, Linder L, Nilsson KG.** A trabecular metal tibial component in total knee replacement in patients younger than 60 years: a two-year radiostereophotogrammetric analysis. J Bone Joint Surg Br 2008; 90: 1585–1593. doi: 10.1302/0301-620x.90b12.20797.

19. **Hilding M, Aspenberg P.** Local peroperative treatment with a bisphosphonate improves the fixation of total knee prostheses: A randomized, double-blind radiostereometric study of 50 patients. Acta Orthop 2007; 78: 795–799. doi: 10.1080/17453670710014572.
20. **Hilding M, Aspenberg P.** Postoperative clodronate decreases prosthetic migration: 4-year follow-up of a randomized radiostereometric study of 50 total knee patients. Acta Orthop 77: 912–916, 2006. doi: 10.1080/17453670610013213.
21. **Hilding M, Ryd L, Toksvig-Larsen S, Aspenberg P.** Clodronate prevents prosthetic migration: A randomized radiostereometric study of 50 total knee patients. Acta Orthop 2000; 71: 553–557. doi: 10.1080/000164700317362163.
22. **Hilding M, Yuan X, Ryd L.** The stability of three different cementless tibial components. A randomized radiostereometric study in 45 knee arthroplasty patients. Acta Orthop 1995; 66: 21–27. doi: 10.3109/17453679508994633.
23. **Hyldahl H, Regnér L, Carlsson L, Kärrholm J, Weidenhielm L.** All-polyethylene vs. metal-backed tibial component in total knee arthroplasty-a randomized RSA study comparing early fixation of horizontally and completely cemented tibial components: part 1. Horizontally cemented components: AP better fixated than MB. Acta Orthop 2005; 76: 769–777. doi: 10.1080/17453670510045354.
24. **Kaptein BL, Hollander P den, Thomassen B, Fiocco M, Nelissen RGHH.** A randomized controlled trial comparing tibial migration of the ATTUNE cemented cruciate-retaining knee prosthesis with the PFC-sigma design. BJJ 2020; 102: 1158–1166. doi: 10.1302/0301-620X.102B9.BJJ-2020-0096.R1.
25. **Kienapfel H, Hildebrand R, Neumann T, Specht R, Koller M, Celik et al**. The effect of Palamed® G bone cement on early migration of tibial components in total knee arthroplasty. Inflammation Research 2004; 53: S159–S163. doi: 10.1007/S00011-004-0362-5.
26. **Koster LA, Meinardi JE, Kaptein BL, Van der Zwaag EV, Nelissen RGHH**. Two-year RSA migration results of symmetrical and asymmetrical tibial components in total knee arthroplasty: a randomized controlled trial. BJJ 2021; 103: 855–863. doi: 10.1302/0301-620X.103B5.BJJ-2020-1575.R2.
27. **Koster LA, Rassir R, Kaptein BL, Sierevelt IN, Schager M, Nolte PA et al.** A randomized controlled trial comparing two-year postoperative femoral and tibial migration of a new and an established cementless rotating platform total knee arthroplasty. Bone Joint J 2023; 105: 148–157. doi: 10.1302/0301-620X.105B2.BJJ-2022-0414.R1.
28. **Laende, EK, Mills Flemming, J, Astephen Wilson, JL, Cantoni, E, & Dunbar, MJ.** The associations of implant and patient factors with migration of the tibial component differ by sex. BJJ 2022; 104-B(4), 444–451. doi:10.1302/0301-620X.104B4.BJJ-2021-1247.R1
29. **Laende EK, Richardson CG, Dunbar MJ.** A randomized controlled trial of tibial component migration with kinematic alignment using patient-specific instrumentation versus mechanical alignment using computer-assisted surgery in total knee arthroplasty. BJJ 2019; 101: 929–940. doi: 10.1302/0301-620X.101B8.BJJ-2018-0755.R3.
30. **Laende EK, Richardson CG, Meldrum AR, Dunbar MJ.** Tibial Component Migration After Total Knee Arthroplasty With High-Viscosity Bone Cement. J Arthroplasty 2021; 36: 2000–2005. doi: 10.1016/j.arth.2021.01.081
31. **Laende EK, Wilson JLA, Flemming JM, Valstar ER, Richardson CG, Dunbar MJ.** Equivalent 2-year stabilization of uncemented tibial component migration despite higher early migration compared with cemented fixation: an RSA study on 360 total knee arthroplasties. Acta Orthop 2019; 90: 172–178. doi: 10.1080/17453674.2018.1562633.
32. **Li MG, Nilsson KG.** The effect of the preoperative bone quality on the fixation of the tibial component in total knee arthroplasty. J Arthroplasty 2000; 15: 744–753. doi: 10.1054/ARTH.2000.6617.
33. **Linde KN, Madsen F, Puhakka KB, Langdahl BL, Saballe K, Krog-Mikkelsen et al.** Preoperative Systemic Bone Quality Does Not Affect Tibial Component Migration in Knee Arthroplasty: A 2-Year Radiostereometric Analysis of a Hundred Consecutive Patients. J Arthroplasty 2019; 34: 2351–2359. doi: 10.1016/j.arth.2019.05.019.
34. **Linde KN, Rytter S, Soballe K, Madsen F, Langdahl B, Stilling M et al.** Component migration, bone mineral density changes, and bone turnover markers in cementless and cemented total knee arthroplasty: a prospective randomized RSA study in 53 patients with 2-year follow-up. Knee Surg Sports Traumatol Arthrosc 2022; 30(9), 3100–3113. doi: 10.1007/s00167-022-06860-4.
35. **Mills K, Wymenga AB, Bénard MR, Kaptein BL, Defoort KC, van Hellemondt GG et al.** Fluoroscopic and radiostereometric analysis of a bicruciate-retaining versus a posterior cruciate-retaining total knee arthroplasty: a randomized controlled trial. Bone Joint J 2023; 105-B: 35–46. doi: 10.1302/0301-620X.105B1.BJJ-2022-0465.R2.

36. **Molt M, Harsten A, Toksvig-Larsen S.** The effect of tourniquet use on fixation quality in cemented total knee arthroplasty a prospective randomized clinical controlled RSA trial. Knee 2014; 21: 396–401. doi: 10.1016/j.knee.2013.10.008.
37. **Molt M, Ljung P, Toksvig-Larsen S.** Does a new knee design perform as well as the design it replaces? Bone Joint Res 2012; 1: 315–323. doi: 10.1302/2046-3758.112.2000064.
38. **Molt M, Toksvig-Larsen S.** 2-year follow-up report on micromotion of a short tibia stem. A prospective, randomized RSA study of 59 patients. Acta Orthop 2015; 86: 594–598. doi: 10.3109/17453674.2015.1033303.
39. **Molt M, Toksvig-Larsen S.** Similar early migration when comparing CR and PS in TriathlonTM TKA: A prospective randomised RSA trial. Knee 2014; 21: 949–954. doi: 10.1016/j.knee.2014.05.012.
40. **Mosegaard SB, Rytter S, Madsen F, Odgaard A, Soballe K, Stilling M et al.** Two-year fixation and ten-year clinical outcomes of total knee arthroplasty inserted with normal-curing bone cement and slow-curing bone cement: A randomized controlled trial in 54 patients. Knee Elsevier 2021;33:110-124. doi: 10.1016/j.knee.2021.08.027.
41. **Nelissen RG, Valstar ER, Rozing PM.** The effect of hydroxyapatite on the micromotion of total knee prostheses. A prospective, randomized, double-blind study. J Bone Joint Surg Am 1998; 80: 1665–1672. doi: 10.2106/00004623-199811000-00014.
42. **Nielsen PT, Berg-Hansen E, Toksvig LS, Ryd L, Rechnagel K, Schroder H et al.** AGC 2000 tibial components with hydroxyapatite coating-a randomized series followed 5 years with RSA. Acta Orthop Scand 1995; Suppl 265-66:77.
43. **Nieuwenhuijse MJ, van der Voort P, Kaptein BL, van der Linden-van der Zwaag HM, Valstar ER, Nelissen RG et al.** Fixation of high-flexion total knee prostheses: five-year follow-up results of a four-arm randomized controlled clinical and roentgen stereophotogrammetric analysis study. J Bone Joint Surg Am 2013; 95: e1411-11. doi: 10.2106/jbjs.l.01523.
44. **Nilsson KG, Dalen T.** Inferior performance of Boneloc® bone cement in total knee arthroplasty: A prospective randomized study comparing Boneloc® with Palacos® using radiostereometry (RSA) in 19 patients. Acta Orthop 1998; 69: 479–483. doi: 10.3109/17453679808997782.
45. **Nilsson KG, Henricson A, Norgren B, Dalén T**. Uncemented HA-coated implant is the optimum fixation for TKA in the young patient. Clin Orthop Relat Res 2006; 448: 129–138. doi: 10.1097/01.BLO.0000224003.33260.74.
46. **Nilsson KG, Kärrholm J, Ekelund L, Magnusson P.** Evaluation of micromotion in cemented vs uncemented knee arthroplasty in osteoarthrosis and rheumatoid arthritis. Randomized study using roentgen stereophotogrammetric analysis. J Arthroplasty 1991; 6: 265–278. doi: 10.1016/s0883-5403(06)80174-9.
47. **Nilsson KG, Kärrholm J.** Increased varus-valgus tilting of screw-fixated knee prostheses. Stereoradiographic study of uncemented versus cemented tibial components. J Arthroplasty 1993; 8: 529–540.
48. **Nivbrant NO, Khan RJK, Fick DP, Haebich S, Smith E.** Cementless Versus Cemented Tibial Fixation in Posterior Stabilized Total Knee Replacement: A Randomized Trial. J Bone Joint Surg Am 2020; 102: 1075–1082. doi: 10.2106/JBJS.19.01010.
49. **Norgren B, Dalén T, Nilsson KG.** All-poly tibial component better than metal-backed: a randomized RSA study. Knee 2004; 11: 189–196. doi: 10.1016/s0968-0160(03)00071-1.
50. **Øhrn FD, Leeuwen J Van, Tsukanaka M, Röhrl S**. A 2-year RSA study of the Vanguard CR total knee system: A randomized controlled trial comparing patient-specific positioning guides with conventional technique. Acta Orthop 2018; 89: 418–424. doi: 10.1080/17453674.2018.1470866.
51. **Önsten I, Nordqvist A, Carlsson å S, Besjakov J, Shott S.** Hydroxyapatite augmentation of the porous coating improves fixation of tibial components. J Bone Joint Surg Br 1998; 80-B: 417–425. doi: 10.1302/0301-620X.80B3.0800417.
52. **Østgaard SE, Dirksen KL LB.** Hydroxyapatite coating in total knee arthroplasty - a randomised RSA study of tibial components. Acta Orthop 1999; 70-Suppl: 4.
53. **Petursson G, Fenstad AM, Gøthesen Ø, Haugan K, Dyrhovden GS, Hallan G et al.** Similar migration in computer-assisted and conventional total knee arthroplasty: A multicenter, parallel-group, randomized controlled trial involving 54 patients. Acta Orthop 2017; 88: 166–172. doi: 10.1080/17453674.2016.1267835.
54. **Regnér L, Carlsson L, Kärrholm J, Herberts P.** Ceramic coating improves tibial component fixation in total knee arthroplasty. J Arthroplasty 1998; 13: 882–889. doi: 10.1016/s0883-5403(98)90194-2.
55. **Regnér L, Carlsson L, Kärrholm J, Herberts P.** Tibial component fixation in porous- and hydroxyapatite-coated total knee arthroplasty: a radiostereo metric evaluation of migration and inducible displacement after 5 years. J Arthroplasty 2000; 15: 681–689. doi: 10.1054/arth.2000.8103.

56. **Ryd L, Albrektsson BE, Herberts P, Lindstrand A, Selvik G.** Micromotion of noncemented Freeman-Samuelson knee prostheses in gonarthrosis. A roentgen-stereophotogrammetric analysis of eight successful cases. Clin Orthop Relat Res 1998; 229, 205–212.
57. **Ryd L, Carlsson L, Herberts P.** Micromotion of a noncemented tibial component with screw fixation: An in vivo roentgen stereophotogrammetric study of the Miller-Galante prosthesis. Clin Orthop Relat Res 1993; 218–225.
58. **Ryd L, Lindstrand A, Rosenquist R, Selvik G.** Micromotion of conventionally cemented all-polyethylene tibial components in total knee replacements. Arch Orthop Trauma Surg 1987; 106: 82–88. doi: 10.1007/BF00435419.
59. **Ryd L, Lindstrand A, Rosenquist R, Selvik G.** Tibial component fixation in knee arthroplasty [Online]. Clin Orthop Relat Res 1986; 141–149.
60. **Ryd, A Lindstrand, A Stenström GS.** Porous coated anatomic tricompartmental tibial components. The relationship between prosthetic position and micromotion. Clin Orthop Relat Res 1990; 251: 97.
61. **Schotanus MGM, Pilot P, Kaptein BL, Draijer WF, Tilman PBJ, Vos R et al.** No difference in terms of radiostereometric analysis between fixed- and mobile-bearing total knee arthroplasty: a randomized, single-blind, controlled trial. Knee Surg Sports Traumatol Arthrosc 2017; 25: 2978–2985. doi: 10.1007/s00167-016-4138-6.
62. **Sporer S, MacLean L, Burger A, Moric M.** Evaluation of a 3D-printed total knee arthroplasty using radiostereometric analysis: Assessment of highly porous biological fixation of the tibial baseplate and metal-backed patellar component. BJJ 2019; 101: 40–47. doi: 10.1302/0301-620X.101B7.BJJ-2018-1466.R1.
63. **Stilling M, Madsen F, Odgaard A, Rømer L, Andersen NT, Rahbek O et al.** Superior fixation of pegged trabecular metal over screw-fixed pegged porous titanium fiber mesh: a randomized clinical RSA study on cementless tibial components. Acta Orthop 2011; 82: 177–186. doi: 10.3109/17453674.2011.566139.
64. **Stilling M, Mechlenburg I, Jepsen CF, Ramer L, Rahbek O, Soballe K et al.** Superior fixation and less periprosthetic stress-shielding of tibial components with a finned stem versus an I-beam block stem: a randomized RSA and DXA study with minimum 5 years follow-up. Acta Orthop 2019; 90: 165–171. doi: 10.1080/17453674.2019.1566510.
65. **Teeter MG, Marsh JD, Howard JL, Vasarhelyi EM, McCalden RW, Naudie DDR et al.** A randomized controlled trial investigating the value of patient-specific instrumentation for total knee arthroplasty in the Canadian healthcare system. BJJ 2019; 101: 565–572. doi: 10.1302/0301-620X.101B5.BJJ-2018-1323.R1.
66. **Teeter MG, Thoren J, Yuan X, McCalden RW, MacDonald SJ, Lanting BA et al.** Migration of a cemented fixed-bearing, polished titanium tibial baseplate (genesis II) at ten years. BJJ 2016; 98: 616–621. doi: 10.1302/0301-620X.98B5.36865.
67. **Tjørnild M, Søballe K, Hansen PM, Holm C, Stilling M.** Mobile- vs. fixed-bearing total knee replacement. Acta Orthop 2015; 86: 208–214. doi: 10.3109/17453674.2014.968476.
68. **Toksvig-Larsen S, Jorn LP, Ryd L, Lindstrand A.** Hydroxyapatite-enhanced tibial prosthetic fixation. Clin Orthop Relat Res 2000; 192–200. doi: 10.1097/00003086-200001000-00018.
69. **Toksvig-Larsen S, Ryd L, Lindstrand A**. Early inducible displacement of tibial components in total knee prostheses inserted with and without cement: a randomized study with roentgen stereophotogrammetric analysis. J Bone Joint Surg Am 1998; 80: 83–89.
70. **Toksvig-Larsen S, Ryd L, Lindstrand A.** Effect of a cooled saw blade on prosthesis fixation: Randomized radiostereometrya of 33 knee cases. Acta Orthop 1994; 65: 533–537. doi: 10.3109/17453679409000908.
71. **Torle J, Thillemann JK, Petersen ET, Madsen F, Soballe K, Stilling M et al.** Less polyethylene wear in monobloc compared to modular ultra-high-molecular-weight-polyethylene inlays in hybrid total knee arthroplasty: A 5-year randomized radiostereometry study. Knee 2021; 29: 486–499. doi: 10.1016/j.knee.2021.02.033.
72. **Troelsen A, Ingelsrud LH, Thomsen MG, Muharemovic O, Otte KS, Husted H et al.** Are There Differences in Micromotion on Radiostereometric Analysis Between Bicruciate and Cruciate-retaining Designs in TKA? A Randomized Controlled Trial. Clin Orthop Relat Res 2020; 478: 2045–2053. doi: 10.1097/CORR.0000000000001077.
73. **Uvehammer J, Kärrholm J, Carlsson L.** Cemented versus hydroxyapatite fixation of the femoral component of the Freeman-Samuelson total knee replacement: a radiostereometric analysis. J Bone Joint Surg Br 2007; 89: 39–44. doi: 10.1302/0301-620x.89b1.17974.
74. **Uvehammer J, Kärrholm J, Regnér L, Carlsson L, Herberts P.** Concave versus posterior-stabilized tibial joint surface in total knee arthroplasty: randomized evaluation of 47 knees. J Arthroplasty 2001; 16: 25–32. doi: 10.1054/arth.2001.17939.

75. **Van der Linde MJ, Grimm B, Garling EH, Valstar ER, Tonino AJ.** Comparative study of the migration of the tibial tray in total knee arthroplasty for cemented, uncoated and periapatite coated components using roentgenstereogrammetric analysis. J Bone Joint Surg Br 2006; 88-B-Suppl, 2006.
76. **Van Der Linde MJA, Garling EH, Valstar ER, Tonino AJ, Nelissen RGHH.** Periapatite may not improve micromotion of knee prostheses in rheumatoid arthritis. Clin Orthop Relat Res 2006; 448: 122–128. doi: 10.1097/01.BLO.0000224005.18013.EE.
77. **van Hamersveld KT Van, Mheen PJM-V De, Tsonaka R, Valstar ER, Toksvig-Larsen S.** Fixation and clinical outcome of uncemented peri-apatite-coated versus cemented total knee arthroplasty: Five-year follow-up of a randomised controlled trial , using radiostereometric analysis (RSA). BJJ 2017; 99: 1467–1476. doi: 10.1302/0301-620X.99B11.BJJ-2016-1347.R3.
78. **Van Hamersveld KT, Marang-van de Mheen PJ, Van Der Heide HJL, Valstar ER, Nelissen RGHH.** Migration and clinical outcome of mobile-bearing versus fixed-bearing single-radius total knee arthroplasty. Acta Orthop 2018; 89: 190–196. doi: 10.1080/17453674.2018.1429108.
79. **van Ooij B, Sierevelt IN, van der Vis HM, Hoornenborg D, Haverkamp D.** What is the role of cemented fixation in total knee arthroplasty? The two-year results of a randomized RSA controlled trial. BJJ 2021; 103-b: 98–104. doi: 10.1302/0301-620x.103b1.bjj-2020-0788.r1.
80. **von Schewelov T, Besjakov J, Sanzén L, Carlsson A**. A clinical and radiostereometric study of the cemented PFC-sigma prosthesis: a 5-year study of 29 cases with a fixed bearing. J Knee Surg 2009; 22: 231–236. doi: 10.1055/s-0030-1247754.
81. **Williams HA, Broberg JS, Howard JL, Lanting BA, Teeter MG.** Effect of gap balancing and measured resection techniques on implant migration and contact kinematics of a cementless total knee arthroplasty. Knee 2021; 31: 86–96. doi: 10.1016/j.knee.2021.05.011.
82. **Winther NS, Jensen CL, Jensen CM, Lind T, Schrøder HM, Flivik G et al.** Comparison of a novel porous titanium construct (Regenerex®) to a well proven porous coated tibial surface in cementless total knee arthroplasty - A prospective randomized RSA study with two-year follow-up. Knee 2016; 23: 1002–1011. doi: 10.1016/J.KNEE.2016.09.010.
83. **Wojtowicz R, Henricson A, Nilsson KG, Crnalic S.** Uncemented monoblock trabecular metal posterior stabilized high-flex total knee arthroplasty: similar pattern of migration to the cruciate-retaining design - a prospective radiostereometric analysis (RSA) and clinical evaluation of 40 patients (49 knees). Acta Orthop 2019; 90: 460–466. doi: 10.1080/17453674.2019.1626097.
84. **Wolterbeek N, Garling EH, Mertens BJ, Nelissen RG, Valstar ER.** Kinematics and early migration in single-radius mobile- and fixed-bearing total knee prostheses. Clinical Biomechanics 2012; 27: 398–402. doi: 10.1016/j.clinbiomech.2011.10.013.
85. **Yilmaz M, Holm C, Lind T, Flivik G, Odgaard A, Petersen M et al.** Bone remodeling and implant migration of uncemented femoral and cemented asymmetrical tibial components in total knee arthroplasty - A DEXA and RSA evaluation with 2-year follow-up. Knee Surg Relat Res 2021; 33. doi: 10.1186/s43019-021-00111-5.
